# Epigenetic Dysregulation in Cancer: Implications for Gene Expression and DNA Repair-Associated Pathways

**DOI:** 10.3390/ijms26136531

**Published:** 2025-07-07

**Authors:** Nina Rembiałkowska, Katarzyna Rekiel, Piotr Urbanowicz, Mateusz Mamala, Karolina Marczuk, Maria Wojtaszek, Marta Żywica, Eivina Radzevičiūtė-Valčiukė, Vitalij Novickij, Julita Kulbacka

**Affiliations:** 1 Department of Molecular and Cellular Biology, Faculty of Pharmacy, Wroclaw Medical University, Borowska 211A, 50-556 Wroclaw, Poland; julita.kulbacka@umw.edu.pl; 2 Faculty of Medicine, Wroclaw Medical University, 50-367 Wroclaw, Poland; katarzyna.rekiel@student.umw.edu.pl (K.R.); piotr.urbanowicz@student.umw.edu.pl (P.U.); mateusz.mamala@student.umw.edu.pl (M.M.); karolina.marczuk@student.umw.edu.pl (K.M.); maria.wojtaszek@student.umw.edu.pl (M.W.); marta.zywica@student.umw.edu.pl (M.Ż.); 3 Department of Immunology and Bioelectrochemistry, State Research Institute Centre for Innovative Medicine, Santariškių 5, 08410 Vilnius, Lithuania; eivina.radzeviciute@imcentras.lt (E.R.-V.); vitalij.novickij@vilniustech.lt (V.N.); 4 Faculty of Electronics, Vilnius Gediminas Technical University, 10223 Vilnius, Lithuania

**Keywords:** epigenetic therapy, DNA repair mechanisms, chromatin remodeling, drug resistance in cancer, non-coding RNA

## Abstract

Epigenetic modifications are heritable, reversible alterations that causally reshape chromatin architecture and thereby influence DNA repair without changing nucleotide sequence. DNA methylation, histone modifications and non-coding RNAs profoundly influence DNA repair mechanisms and genomic stability. Aberrant epigenetic patterns in cancer compromise DNA damage recognition and repair, therefore impairing homologous recombination (HR), non-homologous end joining (NHEJ), and base excision repair (BER) by suppressing key repair genes and lowering access to repair sites. Then it is dissected how loss-of-function mutations in Switch/Sucrose non-fermentable, imitation switch and CHD (Chromodomain helicase DNA-binding) chromatin-remodeling complexes impair nucleosome repositioning, preventing effective damage sensing and assembly of repair machinery. Non-coding RNAs contribute to epigenetic silencing at DNA break sites, exacerbating repair deficiencies. This review evaluates recent advances concerning epigenetic dysfunction and DNA repair impairment. It is also highlighted that nanoparticle-mediated delivery strategies are designed to overcome pharmacologic resistance. It is presented how epigenetic dysregulation of DNA repair can guide more effective and drug-resistant cancer therapies.

## 1. Introduction

**Figure 1:**
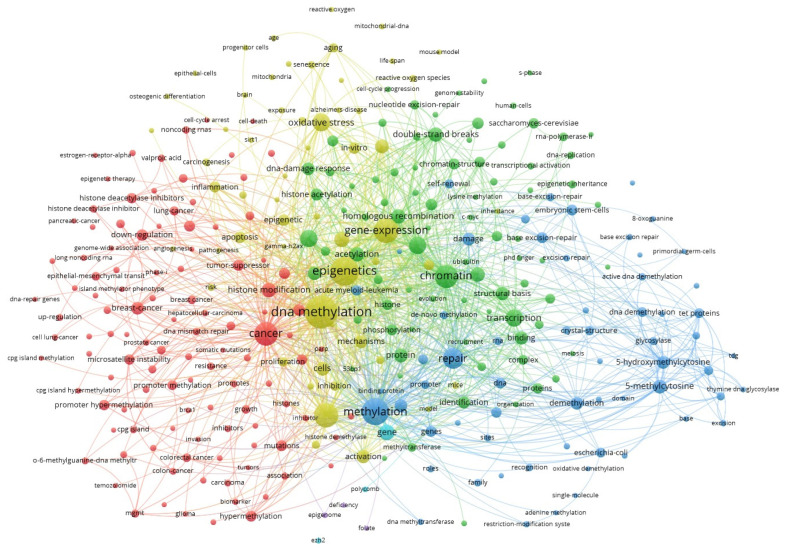
The co-occurrence of keywords in scientific papers processed using VOSviewer, version 1.6.18 (Leiden University). Source: Clarivate Analytics Web of Science; keyword: “epigenetic modifications and DNA repair”; access date: 2 June 2025). Larger circle size corresponds to a higher rate of occurrence of a keyword.

Epigenetic modifications do influence gene expression without altering the underlying DNA sequence, so they can play a role in cancer development and progression. An example of the most frequently co-occurring keywords related to epigenetic modifications and DNA repair is presented in Figure 1, generated using VOSviewer based on data from the Web of Science database. Central nodes such as epigenetics, DNA methylation, cancer, and repair form densely interconnected clusters, reflecting the growing scientific recognition that these processes are not isolated, but part of a highly integrated network. The prominence of terms like chromatin, transcription, and demethylation further underscores the critical role of epigenetic mechanisms in coordinating cellular responses to DNA damage and in maintaining genomic stability. 

This article examines the collective impact of DNA methylation, histone modifications, and chromatin remodeling on genomic stability; specifically, their individual roles are indeed stressed in the particular regulation of DNA damage as well as repair pathways. Dysregulation of epigenetic mechanisms like aberrant promoter methylation with histone acetylation and methylation imbalances, and mutations in chromatin remodeling complexes like SWI/SNF (Switch/Sucrose Non-Fermentable), ISWI (Imitation Switch), and CHD (Chromodomain-Helicase-DNA-binding), can disrupt key DNA repair processes including homologous recombination, non-homologous end joining and base excision repair. The article also explores how non-coding RNAs contribute to epigenetic gene silencing in cancer. We discuss therapeutic strategies that target epigenetic modifications associated with cancer. Current clinical applications and various combination therapies are discussed alongside DNA methyltransferase inhibitors (DNMTis), histone deacetylase inhibitors (HDACi), as well as bromodomain and extra-terminal domain proteins (BET) inhibitors. Emerging approaches, in addition to nanotechnology-based delivery systems, are thoroughly reviewed as resistance mechanisms to epigenetic drugs are addressed. This article is informed by both current research and many published studies throughout the field after a thorough review. Together, these understandings highlight the potential of epigenetic therapies to improve treatment outcomes, and in addition, they provide new avenues for combating resistance in cancer management.

### 1.1. Detailed Mechanisms of DNA Methylation 

An epigenetic process known as DNA methylation creates 5-methylcytosine by adding a methyl group from S-adenosyl methionine (SAM) to the cytosine’s C5 position. DNA methylation primarily occurs at cytosine–phosphate–guanine dinucleotides (CpG, cytosine followed by guanine) [1]. DNA methylation controls gene expression by engaging proteins involved in gene repression or by blocking transcription factors from binding to DNA. Silencing retroviral elements, controlling tissue-specific gene expression, genomic imprinting, and X chromosome inactivation all depend on DNA methylation. Crucially, depending on the underlying genetic sequence, DNA methylation in various genomic locations may have different impacts on gene activity [2].

In normal cells, most CpG dinucleotides are found in repetitive regions, gene bodies, and intergenic regions and are heavily methylated, contributing to genomic stability by suppressing the transcription of potentially harmful sequences such as transposons and other repetitive elements, which, if activated, can lead to genome instability through insertions, deletions, or rearrangements. However, regions with high CpG density, known as CpG islands (CGIs), are often located in the promoters of genes and are typically unmethylated if the genes are expressed. CGIs are often linked to promoters; most tissues tend to express genes whose promoters are particularly rich in CpG sequences. About 70% of gene promoters are found inside CGIs [3]. Promoter CGIs, which are unmethylated in normal tissues, are susceptible to aberrant methylation in pathological conditions, such as cancer. Understanding the dynamics of CGI methylation is pivotal for deciphering gene regulation and disease mechanisms [4]. CGIs have fewer nucleosomes than other DNA segments. Nucleosomes are tiny, packed segments of DNA that are regularly encircled by histone proteins. The DNA’s tolerance for gene expression decreases with its degree of association with histone proteins [5]. 

There was a noticeable tendency toward increased histone H3K9/K14 acetylation and H3K4 trimethylation levels at CpG-island promoters, even though a significant portion of these promoters had decreased histone H3 levels [6]. As a result, it seems that inducible CpG-island promoters are joined together to form chromatin with modifications characteristic of active genes [7]. The process of DNA methylation is catalyzed by a family of enzymes called DNA methyltransferases (DNMT), including DNA (cytosine-5)-methyltransferase 1 (DNMT1), DNA (cytosine-5)-methyltransferase 3 alpha (DNMT3A), and DNA (cytosine-5)-methyltransferase 3 beta (DNMT3B). They possess a comparable structure, featuring a substantial N-terminal regulatory domain and a C-terminal catalytic region; yet they exhibit distinct roles and expression patterns [8]. DNMT1 localizes to the replication fork during DNA replication, which is where synthesized hemimethylated DNA is created. In order to accurately replicate the initial methylation pattern that existed before DNA replication, DNMT1 attaches itself to the freshly produced DNA and methylates it [9,10]. DNMT3A and DNMT3B establish de novo methylation patterns. They are able to give unmodified DNA a new methylation pattern [11]. They have no tendency for hemimethylated DNA and can methylate both synthetic and natural DNA. DNMT3B is expressed weakly, whereas DNMT3A is expressed widely [12]. There is also DNMT3L, which binds to DNMT3A and DNMT3B and increases their methyltransferase activity, but it lacks a catalytic role of its own [13]. 

**Figure 2:**
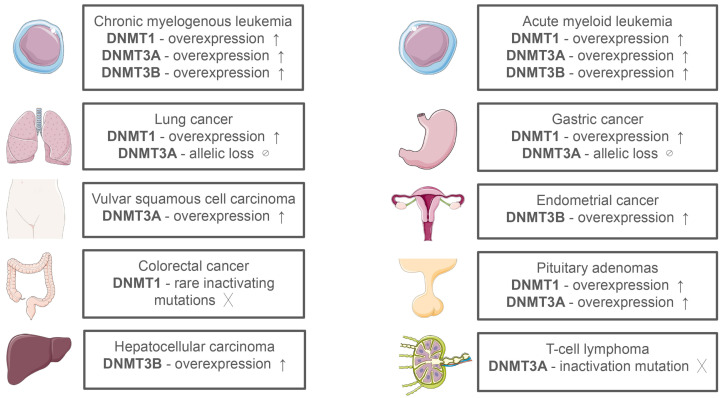
Expression of DNA Methyltransferases in Different Types of Cancer [20].

In cancer, dysregulation of DNMT expression and activity contributes to the altered methylation landscape (Figure 2) [14]. Ten-Eleven Translocation 2 (TET2) is an enzyme that plays a crucial role in epigenetic gene regulation by demethylating DNA. Mutations in the TET2 gene are frequently observed in hematological cancers, such as acute myeloid leukemia (AML), myelodysplastic syndromes (MDS), and chronic myelomonocytic leukemia [15,16]. The DNMT3A mutation is a frequently observed characteristic in the early stages of AML, MDS, and adult T-cell acute lymphoblastic leukemia [16,17]. Co-occurring with DNMT3A mutations, TET2 mutations can be found in the above-mentioned cancers [18]. Certain tumor suppressor genes (TSG) may be transcriptionally silenced as a result of hypermethylation (an increased rate of methylation) of promoter regions, which also helps regulate numerous regulatory proteins and enzymes. On the contrary, a rise of genomic instability and cell transformation is linked to hypomethylation, or a reduced rate of methylation, in the very early stages of cancer development [19].

### 1.2. Histone Modifications

Epigenetic modifications, particularly histone methylation and acetylation, play pivotal roles in the regulation of gene expression and have been extensively studied in the context of cancer. These modifications can lead to either activation or repression of gene transcription, influencing tumorigenesis and cancer progression. Histones are the core proteins around which the 147-base-pair segment of DNA is wrapped, forming nucleosomes, the fundamental units of chromatin. They form an octamer containing the four core histone proteins (H3, H4, H2A, H2B) [21]. Lysine and arginine residues are high in histones, two-thirds of which are concentrated in the tails. Histone-histone and histone-DNA interactions are both facilitated by the carboxyl (C) end of these histones [22]. Post-translational modifications occur at the amino (N) terminal charged tails. The N-terminal tails of histones are subject to a variety of post-translational modifications, including acetylation, methylation, phosphorylation, and ubiquitination [23]. 

#### 1.2.1. Histone Acetylation

Histone acetylation typically occurs on lysine residues of histone tails. It relies on the removal of acetyl groups (CH_3_COO-) from the Acetyl-Coenzyme A (Acetyl-CoA). It is generally associated with transcriptional activation, and it is catalyzed by histone acetyltransferases (HATs) [24]. The four main families of HATs are: p300 and CBP proteins (300 kDa protein and CREB-binding protein); MYST (named for its members, which include MOZ, Ybf2/Sas3, Sas2, and Tip60); Rtt109 (regulator of Ty1 transposition gene production 109); and GCN5 (general control non-repressed protein 5)-related N-acetyltransferases (GNAT) [25].

Histone deacetylases (HDAC), on the other hand, remove the acetyl groups from the acetylated lysine and produce acetate as a byproduct. Based on their catalytic methods, HDACs are divided into two main categories: sirtuins (SIRT), which use the coenzyme nicotinamide adenine dinucleotide (NAD+) as a cofactor, and zinc-dependent (Zn^2+^-dependent) HDACs. Based on structural and functional characteristics, HDACs have also been separated into four classes (I–IV). The similarity of the sequence to the yeast enzymes provides the basis for the division HDACs 1–3 and 8 belong to class I; HDACs 4–7 and 9–10 belong to class II; SIRT1–7, sometimes referred to as SIRT, belongs to class III; and HDAC11 is a member of class IV. Zn^2+^ is required for the activity of HDACs in classes I, II, and IV [26,27]. Both HATs and HDACs are required for properly regulated gene expression [28]. The activity of HAT and HDAC may be regulated by key metabolites, including acetyl-CoA and NAD+, which are involved in protein acetylation and deacetylation processes [29]. The addition of acetyl groups neutralizes the positive charge of lysines, reducing the interaction between histones and negatively charged DNA, leading to a more open and relaxed structure of chromatin conformation (euchromatin) [30]. This change makes it possible for transcription factors and RNA polymerase to attach to the target genes’ promoter region and start transcription [31]. In contrast, histone deacetylation promotes a more condensed chromatin state (heterochromatin) associated with transcriptional repression [32]. 

**Figure 3:**
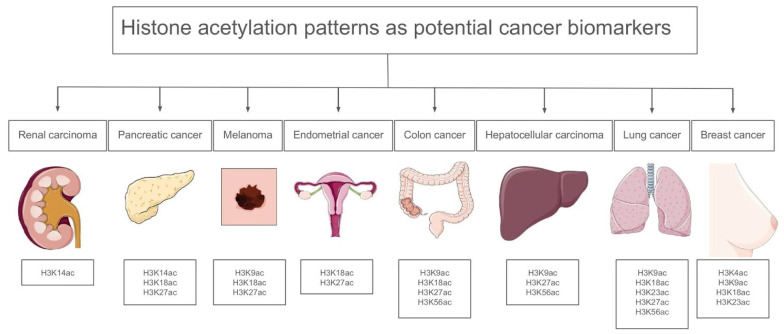
Histone acetylation patterns in specific cancers [38].

Gene expression may be induced by hyperacetylation, particularly in the case of proto-oncogenes, while tumor suppressor genes are often silenced through promoter hypoacetylation, which frequently co-occurs with DNA methylation [33]. Increased HAT activity generally results in chromosomal translocations with various fusion partners and has an overall carcinogenic effect [34]. This enables HATs to acetylate their fusion partner’s genetic targets in an aberrant way. HATs, such as CBP, can contribute to carcinogenesis when they are aberrantly recruited by oncogenic fusion proteins, like MLL-fusion proteins in leukemia, which misdirect their acetyltransferase activity to inappropriate genomic regions, leading to the activation of oncogenes. Overall, the dosage of a HAT determines whether it has tumor-suppressive or carcinogenic effects; overexpression is associated with carcinogenic potential, whereas a decrease in expression leads to acetylation capacity loss [35]. Loss of acetylation at lysine 16 on histone H4 (H4K16ac) is associated with tumorigenesis. This modification is crucial for maintaining chromatin structure and function, and its reduction has been observed in various cancers [36]. HDAC2 directly deacetylates p53 and the CDKN1B/1C/2A proteins in lung cancer, reducing a cell’s capacity to trigger the apoptotic machinery or control the cell cycle [37]. Figure 3 presents representative histone acetylation profiles across selected cancer types, emphasizing site-specific modifications that may serve as potential diagnostic or prognostic indicators.

#### 1.2.2. Histone Methylation

Histone methylation, on the other hand, is a more complex modification with diverse effects on gene expression depending on the specific lysine residue modified and the degree of methylation (mono-, di-, or trimethylation). Histone methylation involves the transfer of methyl groups to the lysine and arginine residues of histone proteins [39]. Histone methyltransferases (HMTs) utilize SAM as a methyl donor to catalyze the methylation process. The methylation can occur in mono-, di-, or trimethyl forms, each imparting distinct functional consequences. They can either contain the SET domain or not [40]. The reversibility of this modification is governed by histone demethylases (HDMs), which remove methyl groups, thereby modulating the chromatin state and gene expression. Lysine-specific demethylase 1 (LSD1) and the Jumonji C (JmjC) domain-containing proteins are prominent examples of HDMs [41]. The JmjC domain is present in KDM4 enzymes, which are responsible for removing methyl groups from histone H3 at lysine 9. They belong to the group of 2-OG-dependent oxygenases and are found to be increased in various types of cancer. Recently, these enzymes have gained significant interest as a new target for cancer treatment [42].

The functional outcome of histone methylation is highly dependent on the specific residue modified and the degree of methylation. Key methylation sites include:

- **H3K4me3** (Histone H3 Lysine 4 Trimethylation): This modification is predominantly associated with transcriptional activation. It marks active promoters and is indicative of genes poised for transcription [43].
- **H3K9me3** (Histone H3 Lysine 9 Trimethylation): Conversely, this mark is linked to transcriptional repression and heterochromatin formation. It plays a crucial role in maintaining genomic stability by silencing transposable elements and repetitive sequences [43].
- **H3K27me3** (Histone H3 Lysine 27 Trimethylation): This modification is mediated by the Polycomb Repressive Complex 2 (PRC2) and is associated with gene silencing during development. It contributes to the establishment of cellular identity by repressing lineage-specific genes in pluripotent cells [44].
- **H3K36me3** (Histone H3 Lysine 36 Trimethylation): This mark is enriched in the coding regions of actively transcribed genes and is implicated in the regulation of alternative splicing and suppression of cryptic transcription initiation [44].
- **H3K4me1** (Histone H3 Lysine 4 Monomethylation) marks poised or active enhancers and plays a role in facilitating enhancer-promoter interactions and transcription [45].
- **H3K4me2** (Histone H3 Lysine 4 Dimethylation) is primarily found at promoters and enhancers and is associated with transcriptionally permissive chromatin. It plays a role in transcription initiation and the maintenance of gene activity, particularly by supporting paused RNA polymerase II and facilitating transcriptional priming [46].
- **H3K36me2** (Histone H3 Lysine 36 Dimethylation) serves as a key epigenetic mark that guides the recruitment of the de novo DNA methyltransferase DNMT3A to intergenic euchromatin, thereby supporting DNA methylation maintenance outside of gene bodies [47].

In cancer, aberrant histone methylation patterns contribute to tumorigenesis by dysregulating oncogenes and TSGs. These alterations often lead to genomic instability, altered cell cycle progression, and evasion of apoptosis. Inactivating mutations in SETD2, the enzyme that adds H3K36me3, are common in kidney cancer and leukemias [48,49]. Nuclear receptor SET domain-containing protein 1 (NSD1) and nuclear receptor SET domain-containing protein 3 (NSD3) are HMTs. NSD1 and nuclear receptor SET domain-containing protein 2 (NSD2) are associated with H3K36me2. Their mutations are commonly found in acute leukemia [50,51]. NSD1 is associated with neuroblastoma [52] and head and neck squamous cell carcinomas [53]. Enhancers of Zeste Homolog 1 (EZH1) and Enhancer of Zeste Homolog 2 (EZH2) are catalytic subunits of the PRC2. They are connected with H3K27me3. Mutations of EZH2 can be found in diffuse large B-cell lymphoma, follicular lymphoma [54,55], and melanoma, whereas mutations of EZH1 are found in thyroid cancer [56]. 

### 1.3. Chromatin Remodeling Complexes

**Figure 4:**
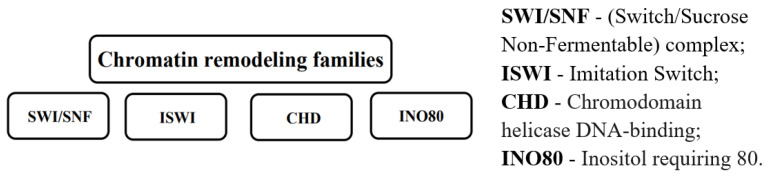
Chromatin remodeling families.

Chromatin remodeling complexes are multi-subunit protein machines that utilize the energy of ATP hydrolysis to alter nucleosome positioning and structure, thereby regulating DNA accessibility and gene expression. These complexes can slide, eject, or restructure nucleosomes, facilitating or inhibiting the binding of transcriptional regulators [57]. In cancer, mutations in chromatin remodeling complexes can cause extensive transcriptional dysregulation. Mutations in remodeling families such as SWI/SNF, ISWI, CHD, and Inositol requiring 80 (INO80) have been linked to oncogenesis, either by promoting TSG suppression or activating oncogenic pathways (Figure 4) [58]. Understanding how chromatin remodeling leads to epigenetic reprogramming in cancer is very important for creating targeted treatments that restore normal chromatin dynamics.

#### 1.3.1. Mechanism of SWI/SNF Action

The SWI/SNF complex, also known as the BAF complex, in a manner consistent with the entire group, is an ATP-dependent chromatin remodeling complex that plays an important role in regulating chromatin structure [59]. It is composed of a number of subunits, such as PBRM1 (Protein Polybromo-1 gene), ARID1A (AT-Rich Interaction Domain Containing protein 1A), ARID1B (AT-Rich Interaction Domain Containing protein 1B), SMARCA4 (SWI/SNF Related, Matrix Associated, Actin Dependent Regulator of Chromatin, Subfamily A, Member 4), and SMARCA2 (SWI/SNF Related, Matrix Associated, Actin Dependent Regulator of Chromatin, Subfamily A, Member 2). Together, these subunits modify chromatin by rearranging, moving, or expelling nucleosomes, which affects gene accessibility [60]. Through modifications to nucleosome orientation, the SWI/SNF complex controls transcription factor binding, promoting either gene activation or repression. It is required for differentiation, DNA repair, and tumor suppression. Mutations in the SWI/SNF complex can cause loss of function, leading to abnormal transcriptional regulation and oncogenesis [61]. 

#### 1.3.2. Impact of SWI/SNF Mutations in Cancer

The tumor-suppressive function of the SWI/SNF chromatin remodeling complex has been recognized in cancers, including ovarian clear cell carcinoma, clear-cell renal cell carcinoma, hepatocellular carcinoma (HCC), gastric cancer, and pancreatic cancer [62]. While most studies have focused on highly mutated subunits like *ARID1A* in ovarian cancer, broader analyses reveal that less frequent mutations in other SWI/SNF subunits also contribute to tumorigenesis in these malignancies [63].

Ovarian clear cell carcinomas are a subtype of ovarian epithelial tumors that develop from the malignant transformation of endometriosis. They are characterized by unique molecular features, high aggressiveness, resistance to standard chemotherapy, and a generally poor prognosis [64]. It is believed that the SWI/SNF complex’s specificity is provided by ARID1A, one of the two ARID1 subunits. Chromatin alterations can affect the epigenetic control of several genes, including those implicated in cancer. Functional studies have linked ARID1A to the SWI/SNF complex’s abilities to suppress cell proliferation [65]. Studies have shown that ARID1A inactivation creates a dependence on glutamine. This correlates with transcriptional repression of the GLS1 gene of the SWI/SNF complex. The inactivation of the SWI/SNF complex changes the metabolic pathway from glycolysis to glutamine dependence. There are studies about pharmacological inhibition of glutaminase, which can be an effective therapeutic strategy for cancers [66]. 

The *SMARCA* genes, part of the SWI/SNF family, are essential for chromatin remodeling and DNA repair [67]. Mutations in *SMARCA2* and *SMARCA4* lead to a loss of their protein expression in the nucleus, contributing to the development of *SMARCA*-deficient cancers. These tumors vary in appearance, ranging from small to large epithelioid cells, giant cells, though rhabdoid cells are not always present. Most of these cancers are high-grade, undifferentiated, or dedifferentiated pleomorphic carcinomas, with some areas showing better differentiation [68]. Studies on lung cancer have identified *SMARCA4* alterations as significant contributors to disease progression. The most common alteration, classified as Class I, consists of truncating mutations that lead to the loss of *SMARCA4* expression. In contrast, Class II alterations involve missense mutations. Notably, Class I alterations are among the most adverse prognostic factors, being strongly associated with reduced survival [69].

Pancreatic cancer remains a leading cause of cancer-related deaths, with survival rates stagnantly low. The near equivalence of incidence and mortality underscores the limitations of current treatments. Epigenetic mechanisms influencing DNA damage and repair could reveal novel therapeutic strategies [70]. ARID1B, a potential DNA-binding subunit of the SWI/SNF chromatin remodeling complex, maintains genomic stability [59]. Studies have identified focal homozygous deletions of ARID1B in a small subset of cases, while broader single-copy deletions affecting this gene were observed in 74% of other samples. These findings suggest a significant role for ARID1B loss in disrupting chromatin structure and DNA repair mechanisms in pancreatic cancer [62]. Mutations or deletions affecting SWI/SNF complex subunits are present in approximately 20–25% of human cancers, including lung cancer, ovarian cancer, and rhabdoid tumors. These genetic alterations often lead to disruptions in chromatin remodeling, resulting in the silencing of TSGs or the activation of oncogenic pathways.

#### 1.3.3. ISWI Chromatin Remodeling Complexes

The mammalian ISWI genes, *SMARCA1* and *SMARCA5*, encode the ATP-dependent chromatin remodeling proteins SNF2L and SNF2H, which are involved in regulating chromatin structure and gene expression as they belong to the group of chromatin remodeling complexes [71]. ISWI remodelers organize chromatin by adjusting the spacing between nucleosomes, which is essential for keeping genes accessible to the cell’s regulatory machinery. This ensures that transcription factors and other proteins can reach the DNA when needed. Beyond regulating gene expression, ISWI complexes also assist in DNA replication by repositioning nucleosomes after the DNA is copied. Additionally, they support DNA repair by making damaged areas more accessible to the repair machinery, helping maintain genome stability [72]. 

#### 1.3.4. ISWI Chromatin Remodeling Complexes in Cancer

Disordered chromatin remodeling contributes to tumorigenesis, with the ISWI family of ATP-dependent chromatin remodelers involved in gene regulation. ISWI complexes are evolutionarily conserved and necessary for cellular function through genetic and epigenetic mechanisms [73]. Omics sequencing and clinical studies have identified widespread ISWI gene expression abnormalities in cancers, linking them to patient outcomes and drug responses. Functional alterations in ISWI-containing complexes influence tumor initiation and progression, while ISWI-associated non-coding RNA networks further shape cancer pathophysiology [74]. Due to their role in transcriptional regulation, ISWI complexes are being explored as potential targets for cancer therapy [75]. 

#### 1.3.5. Chromodomain-Helicase-DNA-Binding Family 

The next important family is the CHD chromatin remodeling complexes. This family consists of a subfamily I (CHD1, CHD2), subfamily II (CHD3, CHD 4, CHD5) and subfamily III (CHD6, CHD7, CHD8, CHD9) [75]. A common feature of CHDs is having double chromodomains at their N-terminal region and a central SNF2 helicase-like ATPase domain. Belonging to the group of chromatin remodeling complexes, members of the CHD family act as ATP-dependent chromatin remodelers, exerting their function by reorganizing nucleosome positioning, controlling DNA accessibility, and contributing to the preservation of genome integrity [76]. CHD proteins have limited DNA sequence specificity, so their association with chromatin is provided by interactions with transcription factors, modified histones, RNA, poly (ADP-ribose), and methylated DNA [77]. It is presumed that CHD recruitment to chromatin occurs step-by-step, with specific transcription factors or protein complexes guiding their targeting to chromatin sites [78]. 

CHD4 is an epigenetic regulator linked to breast cancer and is being explored as a potential therapeutic target. As a core component of the nucleosome remodeling and deacetylase complex (NuRD) [79], CHD4 plays a role in chromatin modification and gene regulation [80]. Mutations in *CHD4* have been identified in breast cancer patients, affecting its ATPase subunit (Mi-2β), the primary catalytic unit of NuRD, which also includes histone deacetylases HDAC1 and HDAC2 [81]. Epigenetic mechanisms involving CHD4 contribute to tumor development, cellular plasticity, and tumor heterogeneity. Certain mutations (*R1162W*, *H1196Y*, *H115R*, and *L1215P*) may alter or reduce CHD4 function, potentially promoting oncogenic transformation [82]. 

#### 1.3.6. INO80

As a member of the chromatin remodeling complexes, INO80 fulfills its function by utilizing ATP-dependent nucleosome repositioning to regulate DNA accessibility, thereby contributing to key cellular processes such as DNA repair, replication, and genomic stability. Its own characteristic is composed of several subunits, including the INO80 ATPase and the RUVBL1/2 helicases, which coordinate to remodel chromatin in response to cellular signals. Dysregulation of INO80 function has been implicated in lung cancer. In tumors, both overexpression and mutations of INO80 subunits have been observed, contributing to transcriptional deregulation of genes involved in cell proliferation and survival [83].

### 1.4. BET Proteins 

The Bromodomain and extra-terminal domain (BET) protein family consists of four proteins: BRD2, BRD3, BRD4, and BRDT, which play a crucial role in regulating transcription. Distinguished by two N-terminal acetyl-lysine binding bromodomains (BD1 and BD2) and an ET domain [84]. The BD1 and BD2 domains identify acetylated lysine in histones or transcription factors and interpret acetylation signals in gene transcription within chromatin. The ET domain guarantees transcriptional activation by attracting transcription regulatory proteins. BRDT is expressed only in the testes, while the rest of the BET family proteins are expressed in other tissues of the body [85]. The most established function of BET proteins, especially BRD4, is the role in transcriptional elongation. BRD4 is most enriched in super-enhancers that promote the expression of tumor growth and development-enhancing factors, including c-MYC [86]. Besides their role in gene transcriptional elongation, recent technological discoveries have led to other functions of BET proteins, including chromatin organization and remodeling, super-enhancer assembly and condensate formation, DDR, and genome integrity. Upregulation of BET proteins is repeatedly seen in cancer to increase oncogenic activity [84]. BET-BD inhibitors have demonstrated therapeutic efficiency in preclinical models. Nevertheless, the function of BRD4 in the regular cell development process and the subsequent prolonged BET-BD inhibition in normal tissue toxicity has been reported in the clinical assessment of distinct BET-BD inhibitors, although it has not been well described [84,87].

### 1.5. Epigenetic Alterations of Key DNA Repair Genes 

Lynch syndrome, also referred to as hereditary nonpolyposis colorectal cancer, is a genetic condition responsible for around 3% of all colorectal cancer cases. Among individuals with Lynch syndrome, colorectal and endometrial cancers are the most commonly diagnosed tumors. A key molecular event in the development of these cancers is the epigenetic silencing of the MLH1 gene through promoter hypermethylation. This modification suppresses gene transcription, leading to the absence of MLH1 protein and the emergence of microsatellite instability, a hallmark of tumors arising in this context [88].

Hypermethylation of cytosine–guanine islands within the 5′ regulatory region of the BRCA1 gene leads to transcriptional repression and diminished BRCA1 protein levels, which in turn impairs homologous recombination-mediated DNA repair. Such alterations are frequently observed in sporadic cases of breast cancer, with a notable prevalence in triple-negative breast cancers [89].

The post-translational modification of histone H2B by monoubiquitination at lysine 120 plays a multifaceted role in regulating chromatin dynamics. It is critically involved in processes such as transcriptional elongation, coordination of histone modifications, centromere integrity, DNA damage response, and the proper maturation of replication-dependent histone mRNAs. Additionally, it is essential for guiding stem cell differentiation. A widespread reduction or loss of this modification has been observed in various high-grade cancers, where it often correlates with enhanced tumor aggressiveness and unfavorable clinical outcomes [90,91].

## 2. Impact on DNA Repair Processes

### 2.1. Specific Epigenetic Changes Leading to Gene Silencing

Epigenetic changes are very significant in pathogenesis. The most important are DNA methylation, histone modification, and changes in chromatin structure. Diagnosis and treatment are facilitated by next-generation sequencing methods and bioinformatics analysis [92]. 

Epigenetic changes play a crucial role in DNA damage response and repair by modifying chromatin structure. The purpose of gene silencing is to regulate gene expression [93]. Epigenetic gene silencing refers to gene inactivation without mutations that can be conscientiously propagated from precursor cells to their daughter cell clones. Promotor CpG Island Hypermethylation is a hallmark of cancer, leading to transcriptional repression of tumor-suppressor genes. DNA Methyltransferases (DNMT1, DNMT3a/3b) contribute to aberrant methylation patterns in cancer. Methyl-CpG Binding Proteins (MBDs): MeCP2 (Methyl-CpG-binding protein 2), MBD1 (Methyl-CpG-binding domain protein 1), MBD2 (Methyl-CpG-binding domain protein 2), and MBD3 (Methyl-CpG-binding domain protein 3) bind to methylated CpG sites, recruiting histone-modifying enzymes and chromatin remodelers [94]. 

Small interfering RNA (siRNA) can cause the division of mRNA molecules, leading to their destruction. Targeted gene silencing is a result of this process, caused by a nano-delivery system. Nanoparticles have a major impact on inhibiting tumor growth because they enable effective transport of the drug and small non-coding RNA to cancer cells, which in turn allows for the reduction of the side effects of chemotherapy [95]. Genome-wide trends of DNA and histone modifications are formed during early development and are sustained through numerous cell divisions. In cancer, the typical epigenetic patterns are altered, leading to the activation of anti-apoptotic and pro-proliferative genes while silencing TSGs such as CDKN2A (Cyclin-Dependent Kinase Inhibitor 2A) [96].

One of the epigenetic processes is gene silencing linked to non-coding RNA. Non-coding RNA (ncRNA) is an active RNA molecule that is transcribed but not converted into proteins. Recent research indicates that ncRNA molecules are crucial in epigenetic gene expression and are probably accountable for significant variations in phenotype among species and in human populations [97]. 

Noteworthy ncRNA molecules include microRNAs (miRNAs) and small interfering RNAs (siRNAs), both of which consist of fewer than 30 nucleotides, as well as long non-coding RNAs (lncRNAs), which contain 200 or more nucleotides. Research has demonstrated that siRNAs and lncRNAs influence gene expression by creating heterochromatin [98]. 

Changes in ncRNA regulation affect cell cycle progression, the level of cancer cell aggression, and metastasis. The significant impact of ncRNA deregulation occurs in cancers, including multiple myeloma (MM). One type of ncRNA—IncRNA—might be used as a marker in making a diagnosis and treatment of MM, according to some studies [99]. ncRNAs have the ability to synthesize bioactive peptides encoded by small open reading frames (sORFs). These peptides can inhibit the proliferation of tumor cells. One of them, MAPK1-109 aa, contributes to reduced levels of phosphorylation of MAPK1 and suppression of tumor proliferation [100]. Only some of the sORFs within ncRNAs have peptide- or protein-coding ability. The most important of them are the peptides encoded by lncRNA, proteins or peptides by circRNA, and proteins and peptides by pri-miRNA. The most important among them are the peptides encoded by IncRNAs, circRNAs, and pro-miRNAs. They are substantial because of their major influence on the glucose metabolism regulation, the epithelial-to-mesenchymal transition, and the ubiquitination pathway. Thereby, they determine a cell’s fate [101]. 

IncRNAs bind epigenetic modifiers to specific genomic regions in HCC (hepatocellular carcinoma). This affects the regulation of gene transcription and leads to demethylation or hypermethylation of DNA. This indicates that epigenetic regulation by IncRNAs can be used as a treatment method to help patients who suffer from this type of cancer [102]. The HOXB-AS3 peptide, encoded by lncRNA HOXB-AS3, suppresses CRC cell growth (it refers to the proliferation or growth of Colorectal Cancer cells) and regulates the metabolism of cells that form tumors. They manage to do that due to the enormous impact on colony formation, migration, invasion, and tumorigenesis [103].

### 2.2. Changes in Chromatin Structure 

#### 2.2.1. Changes in Chromatin Structure Affecting HR, NHEJ, and BER 

##### Homologous Recombination (HR) 

Homologous recombination (HR) involves two similar or identical molecules of DNA exchanging nucleotide sequences. Double-strand break repair and DNA repair depend on this process, hence preserving cellular activities. But some malignancies—breast, ovarian, endometrial, pancreatic, and prostate cancers, among others—may lose this route [104,105]. Carcinogenesis consists of many steps, and tumors often have several mutations that influence cell death, genome stability, and cell development. Mechanisms that signal DNA damage and DNA repair control the stability of genetic material in cells. By enabling precise DNA repair via HR, both BRCA1 and BRCA2 proteins are vital for maintaining genomic stability. Damaged BRCA capabilities cause genomic instability and turn healthy cells into tumor-initiating cells or CSCs (cancer stem cells), hence fostering further tumor development. On chromosome 17 (17q21, base pairs 43,044,294 to 43,125,482), BRCA1 can be found, containing 1863 amino acids and 24 exons. BRCA2 consists of 3418 amino acids [106].

BRCA1 is a protein with various important regions essential for its functioning. The RING (Really Interesting New Gene) domain at the beginning allows it to engage with BARD1 (BRCA1 Associated RING Domain protein 1), creating an E3 ubiquitin ligase complex. The BRCT (BRCA1 C-terminal) domains found at the end attach to phosphopeptides and collaborate with proteins such as CtIP, ABRAXAS (BRCA1 A Complex Subunit), and BRIP1 (also known as FANCJ, BACH1, or BRCA1-interacting protein 1). The middle section, spanning exons 11–13, contains two signals for nuclear localization and a coiled-coil domain that helps BRCA2 link with PALB2 (partner and localizer of BRCA2). Changes in this region are frequently associated with breast cancer [107].

##### Non-Homologous End Joining (NHEJ) 

NHEJ stands for Non-Homologous End Joining, which is a DNA repair pathway used by cells to fix double-strand breaks (DSBs) in DNA. cNHEJ (classical non-homologous end joining) and HR take part in the repair of double-stranded DNA breaks throughout the cell cycle. Both of these pathways are active in the S and G2 stages. Homologous recombination is suppressed in the G1 phase. Joining of unprotected telomeres, which typically occurs via cNHEJ, is restricted to the G1 phase. Upon detecting DNA breaks, the cNHEJ pathway reconnects them without sequence specificity, which may result in genetic damage. HR relies on the production of three overhangs that interact with comparable sister chromatids to help accurate break repair. The stage of the cell cycle and the characteristics of the break determine the choice between HR and cNHEJ [108].

##### Base Excision Repair (BER) 

The major classes of endogenous DNA damage, including alkylations, oxidations, deaminations, depurination, and single-strand breaks (SSBs), are all repaired by the highly conserved base excision repair (BER) pathway. A DNA glycosylase starts the process by identifying and eliminating the damaged base, resulting in an abasic site. After this, a typical route uses an AP-endonuclease (APE) to create a 3′ OH end at the site of the lesion, then a DNA polymerase performs repair synthesis, and a DNA ligase seals the nick. This route is also in charge of immediately repairing DNA single-strand breaks with blocked ends that are brought on by ROS. Remarkably, the damaged bases are quickly found in a large excess of healthy bases, without any energy input. The nucleus and mitochondria are where BER occurs; it protects against cancer, neurodegeneration, and aging [109,110].

#### 2.2.2. Changes in Chromatin Structure and DNA Repair Pathways in Cancer

##### Chromatin Remodeling and Oncogenic Rewiring

The genomic DNA is packaged into a condensed structure, the chromatin. It is composed of DNA wrapped around histone proteins. Due to its significant role in many cellular processes, it is crucial in controlling transcription, replication, and DNA repair by many post-translational modifications of histones [111]. Higher-order chromatin structure is very significant in cancer, as its variants may lead to modification of gene expression. Changes in 3D genome structure that involve rearrangement in enhancer–promoter communication are perceived as a mechanism for activation of oncogenes in cancer. If the rearrangements between TADs (topologically associating domains) concern particular loci (MYC, TERT, CCND1 genes), the prognosis is considerably worse [112]. 

##### DNA Double-Strand Breaks

We regard double-stranded DNA breaks (DSBs) as the most serious damage since they need to be repaired to preserve genome stability, which is critical for cell survival. Improperly repaired or not repaired, DSBs might cause tumors or even cellular death. Homologous recombination (HR) and non-homologous end joining (NHEJ) are the main techniques used to repair DSBs in humans [113].

The pathological DSBs are formed by reactive oxygen species, DNA replication errors, ionizing radiation, and unintentional cleavage by nuclear enzymes. The non-homologous DNA end joining (NHEJ) pathway is especially responsible for the DSBs repair process. The NHEJ pathway uses proteins that recognize, resect, polymerize, and ligate the DNA ends. The process is flexible, which leads to mutations in repaired DNA junctions [114]. NHEJ directly reconnects chromosome ends, and therefore is not subject to the same necessity for both homology (to serve as a template) and extensive synthesis, in contrast to HR. This enables NHEJ to be accessible for repair at any point during the cell cycle [115]. NHEJ is rapid in terms of kinetics and efficient in terms of energy. NHEJ repair starts instantaneously after the occurrence of a DNA double-strand break and can be observed within 30 min of creating a site-specific DSB. In reaction to DNA double-strand breaks, the Ku70-Ku80 heterodimer effectively attaches to the DNA ends and encourages the recruitment of DNA-PKcs (DNA-dependent protein kinase catalytic subunit) [116]. Due to the inability of NHEJ to utilize redundancy, this pathway will have its drawbacks. Therefore, repair products typically involve deletion because the standard DNA transactions—synthesis and ligation—need to be made more flexible [117]. Impairment of NHEJ function can result from the complexity of the DNA double-strand break, including chemically modified or incompatible ends that hinder proper repair. Difficulty in the functioning of NHEJ will also be connected to the multitude of potential damages present in the DSB. Among these, we can identify intermediates in V(D)J recombination featuring hairpin gaps, mismatches, and gaps that might occur when pairing ends together [118]. NHEJ will employ several factors that recognize DSBs, align ends, and serve as a scaffold for various processing factors that aid in the elimination (and occasionally even substitution) of ligation-blocking lesions. Owing to the range of strategies utilized by NHEJ, it will be feasible to mend DSBs with a diversity of end structures. Due to the mechanistic flexibility of NHEJ, it is capable of repairing DNA double-strand breaks with various types of DNA end configurations, including blunt ends, overhangs, or damaged termini [119]. 

##### Homologous Recombination, BRCA Genes, and Therapeutic Targeting 

Using a template for accuracy, homologous recombination (HR) repairs double-strand breaks (DSBs). In cells dividing mitotically, HR mostly depends on the replicated sister chromatid as a specific repair template throughout the S and G2 phases. Called synthesis-dependent strand annealing (SDSA), this unique HR method guarantees high fidelity. HR is more precise than non-homologous end joining (NHEJ) but demands more energy, works more slowly, and usually takes 6–24 h for completion [120]. HR is a DNA repair mechanism that relies on recombinase RecA in prokaryotes and recombinase Rad51 in eukaryotes. It repairs damage such as DSBs and single-stranded DNA gaps (ssDNA gaps). HR is involved in the DNA replication process, protecting replication forks. HR increases the level of tolerance of eukaryotic cells to replication stress. It plays a major role in anti-cancer therapy [120]. RecA and Rad51 proteins usually bind ssDNA, which requires them to bind ATP. Rad51 plays a significant role in homology search and creating a three-stranded paranemic intermediate. The target in this process is the sister chromatid. The Rad51 pathway is not active in the G1 phase of the mitotic cycle [121]. Due to the resection of DSB ends, ssDNA becomes exposed with 3′ ends, and Rad51 fibers are formed. BRCA2 (Breast Cancer 2) is a very important gene when it comes to the integrity of DNA, as it loads Rad51 into single-stranded DNA regions. It is vital at the beginning of the strand invasion process during HR. Its mutation may lead to ovarian and breast cancers [122]. However, mutations in BER genes can weaken DNA repair, compromising genomic integrity [123]. Cancer cells rely on increased BER activity to survive oxidative stress, which also reduces the effectiveness of radiotherapy and chemotherapy by repairing treatment-induced DNA damage. Targeting BER can enhance cancer treatment by promoting the accumulation of unrepaired single-strand breaks (SSBs), which can convert into double-strand breaks (DSBs) during replication. The resulting DSBs are highly cytotoxic and can lead to replication fork collapse, genomic instability, and ultimately cell death [124]. Targeting BER can enhance cancer treatment by overwhelming cancer cells with DNA damage, leading to stalled replication and lethal DSB accumulation. Since cancer cells divide faster than normal cells, they are more vulnerable to BER inhibition, while normal cells can recover post-treatment [124].

By promoting DNA end resection and recruiting the RAD51 recombinase complex, BRCA1—along with BRCA2—plays a crucial role in homologous recombination [125,126]. Its role is critical to the initial stages of double-strand break repair, functioning upstream of BRCA2. Cells become very reliant on alternative, error-prone repair pathways like non-homologous end joining (NHEJ) when there is a deficiency in BRCA1 or BRCA2. PARP inhibitors are used therapeutically to target this vulnerability by preventing single-strand breaks from being repaired, which causes synthetic lethality in tumors lacking BRCA [127,128]. Furthermore, novel methods include suppressing NHEJ to make BRCA1/2-deficient cells more susceptible to DNA-damaging agents [105].

## 3. Therapeutic Approaches Targeting Epigenetic Changes 

### 3.1. Novel Epigenetic Drugs 

Epigenetic alterations contribute not only to the distinctive properties of cancer cells but also to the challenges associated with their treatment. One major obstacle is cancer cell heterogeneity and the development of drug resistance [129]. Drug resistance can arise from epigenetic mechanisms such as DNA methylation, non-coding RNA modifications, and histone acetylation and methylation. Current research focuses on broadening our understanding of the inhibitors targeting these epigenetic regulators [130]. Scientists are actively working on the discovery of novel epigenetic drugs. In this section, we summarize current knowledge in this field. Table 1 summarizes clinically approved HDAC and DNMT inhibitors along with their cancer indications, approval timelines, and reported efficacy profiles.

#### 3.1.1. Histone Deacetylases Inhibitors 

Histone deacetylases are one of the regulators of epigenetic changes. The process of HDAC involves the removal of an acetyl group from lysine residues on target proteins, which leads to chromatin remodeling and regulates various cellular processes. HDAC inhibitors are responsible for loosening the chromatin structure of the tumor suppressor gene, resulting in suppression of the tumor. Moreover, these inhibitors induce apoptosis, regulate cell-cycle arrest, and increase the sensitivity of cancer cells to treatment [131]. HDAC inhibitors are categorized into several types, including hydroxamic acid derivatives, benzamides, cyclic peptides, carboxylic acid derivatives, and ketones. As of today, we have a few drugs approved by the United States Food and Drug Administration (FDA) to treat specific types of cancer. FDA-approved HDAC inhibitors are Vorinostat, Romidepsin, Panobinostat, and Belinostat [132]. The Tucidinostat received approval from the National Medical Product Administration (NMPA) of China [133]. The rest are under clinical investigations, including Abexinostat, Fimepinostat, Trichostatin A, Entinostat, KA2507, OBP-801, and Givinostat, among many others [133].

(A) Vorinostat

Vorinostat (SAHA) was the first histone deacetylase inhibitor (HDACi) to enter the market. SAHA belongs to the hydroxamic acid HDAC inhibitors. It enhances protein acetylation, regulates gene expression, and triggers processes such as differentiation, growth arrest, and apoptosis in tumor cells. This drug works by inhibiting class 1 and class 2 HDAC enzymes through binding to their active sites. This mechanism may prove beneficial for specific hematological cancers, especially those associated with class 1 HDAC enzymes. In 2006, the United States FDA approved SAHA for the treatment of cutaneous T-cell lymphoma (CTCL) [134].

The approval was based on a phase II trial in which patients with CTCL received 400 mg Vorinostat orally per day; the results showed an objective response at 29.7% overall, and the average treatment duration was 5.3 months. The majority of adverse effects were mild to moderate in severity and did not necessitate any reduction in dosage or interruption of treatment. Of the 74 patients, 21 (28%) experienced drug-related grade 3 or 4 adverse events. The most frequently reported issues included fatigue (5%), pulmonary embolism (5%), thrombocytopenia (5%), and nausea (4%) [135]. SAHA has proven to be effective in CTLC therapy, but the outcomes of clinical trials involving its use as a monotherapy for solid tumors have not been particularly promising. In an initial phase II clinical trial, SAHA was given to patients suffering from relapsed or refractory breast, colorectal, or non-small cell lung cancer (NSCLC). Unfortunately, the trial was halted prematurely because of considerable reported toxicities and the lack of any patients showing a partial response (PR) or complete response (CR) as defined by the Response Evaluation Criteria in Solid Tumors (RECIST) [136,137].

Vorinostat has been studied in clinical trials for the treatment of breast cancer (BC), both as monotherapy and in combination therapy. Current studies are focused on the treatment of breast cancer with Vorinostat in combination therapy with chemotherapeutics, such as Cisplatin. SAHA, whether used alone or in combination with other strategies, has demonstrated promising activity in BC. Vorinostat’s effects have not been approved because further research is essential to evaluate its effectiveness and determine the optimal doses and treatment regimens [138]. Vorinostat effectively inhibits both the proliferation and migration of cervical cancer cell lines while promoting apoptosis and inducing S-phase cell cycle arrest in a manner that was both dose- and time-dependent. Additionally, Vorinostat increases the expression of major histocompatibility complex class I-related chain A (MICA) through the PI3K/Akt signaling pathway, thereby enhancing the ability of natural killer (NK) cells to target and eliminate tumor cells. This could provide a basis for further research into Vorinostat as a future treatment for cervical cancer [137].

(B) Romidepsin

Romidepsin is another approved HDACi and belongs to the class of cyclic peptide inhibitors. It serves as a powerful inhibitor of HDAC activity, specifically targeting class I and II HDAC enzymes. Romidepsin received FDA approval in 2009 for the treatment of CTCL and subsequently in 2011 for the treatment of peripheral T-cell lymphoma (PTCL) [132,134]. The US FDA approved the effects of Romidepsin for CTCL patients based on a phase II trial in which the patients received Romidepsin in an intravenous infusion at 14 mg/m^2^ on 1, 8, and 15 days of 28-day cycle, the overall response rates (ORR) were 34% and 35%, while the complete response rate (CR) was at 6% in both studies. Romidepsin provides a duration of response lasting between 11 and 15 months. The most frequently reported drug-related adverse events were nausea and fatigue, which were classified as grade 2 or 3 [139,140,141].

Romidepsin also received approval for the treatment of PTCL by the FDA based on results of a phase II study, in which patients who had not responded to at least one prior systemic therapy, or for whom at least one systemic treatment had been unsuccessful, were administered Romidepsin at a dose of 14 mg/m^2^. This was given as an intravenous infusion over four hours on days 1, 8, and 15, with a treatment cycle every 28 days. The phase II study involved 130 patients. The overall response rate (ORR) reached 25%, with 15% of patients achieving a complete response (CR). Among the most frequently observed grade ≥3 drug-related adverse events were thrombocytopenia at 23%, neutropenia at 18%, and infections at 6% [142]. 

Romidepsin, similar to Vorinostat, shows low efficacy in monotherapy treatment of solid tumors. Romidepsin showed only 7% objective response in the treatment of refractory metastatic renal cell cancer and additionally caused many side effects, including hematologic, gastrointestinal, and cardiac toxicity. The treatment of metastatic colorectal cancer with the use of Romidepsin was stopped due to no objective responses and adverse effects [143].

(C) Belinostat

The third drug approved by the US FDA in July 2014 for treating relapsed or refractory peripheral T-cell lymphoma (PTCL) was Belinostat. Belinostat belongs to the class of hydroxamic acid HDAC inhibitors. It functions by inhibiting zinc-dependent deacetylase enzymes from classes I, II, and IV, with a primary focus on ecto-HDAC1, HDAC2, HDAC3, and HDAC6 [134,144].

The approval was based on the results of a phase II study that involved 129 individuals with PTCL who had either not responded to treatment or had relapsed. In this study, belinostat was administered intravenously at a dosage of 1000 mg/m^2^ on days 1 to 5 of a 21-day cycle. Among the 120 patients evaluated, the overall response rate (ORR) was reported to be 25.8%, with 10.8% achieved a complete response and 15% achieved a partial response [132]. Belinostat treatment was well tolerated, as the majority of patients (113 out of 129, or 87.6%) successfully maintained the target dose of 1000 mg/m^2^. The most frequently reported grade 3 to 4 adverse events included anemia, occurring in 10.8% of cases; thrombocytopenia, affecting 7%; and both dyspnea and neutropenia, each observed in 6.2% of patients [145].

Belinostat is an example of another HDAC inhibitor that shows low efficacy in the treatment of solid tumors, including malignant pleural mesothelioma, ovarian cancer, and both thymoma and thymic carcinoma [145,146,147].

(D) Panobinostat

Another example of an HDAC inhibitor is Panobinostat, which was approved by the US Food and Drug Administration in 2015 and belongs to the hydroxamic acid inhibitor class. Panobinostat targets class I, II, and IV HDAC enzymes. It primarily affects HDAC1, 2, 3, and 6, and plays a significant role in cancer development [132,134].

Panobinostat has been proven by the FDA to be effective in the treatment of MM in combination with bortezomib and dexamethasone. Patients who received therapy with panobinostat-bortezomib-dexamethasone exhibited a higher frequency of achieving deeper responses, such as very good partial response (VGPR) or CR, with rates of 28% compared to 16% for those receiving placebo-bortezomib-dexamethasone therapy. The objective response was at 58.5% [148]. Side effects experienced by patients treated with Panobinostat include hematologic toxicity, cardiotoxicity, and diarrhea [149].

Early results from clinical trials showed low effectiveness of Panobinostat in the treatment of solid tumors. These results in the treatment of solid tumors are similar to those of other HDAC inhibitors, including Vorinostat and Romidepsin. The four approved HDAC inhibitors are indicated for hematological cancers, including CTCL, PTCL, and MM. However, they are not approved for the treatment of solid tumors [144].

However, the results of the phase II study showed the safety and activity of Panobinostat in patients with relapsed or refractory Hodgkin’s lymphoma (HL) after autologous stem-cell transplantation (ASCT). A total of 129 patients participated in the study; 35 patients (27%) experienced an objective response, consisting of 30 patients (23%) with partial responses and five patients (4%) with complete responses. The median time to response (TTR) was 2.3 months, the median duration of response (DOR) was 6.9 months, and the median progression-free survival (PFS) was 6.1 months. The most frequently observed grade 3 and 4 hematological adverse events include thrombocytopenia, anemia, and neutropenia [150].

(E) Tucidinostat

Tucidinostat is a new, orally administered drug from the benzamide class, functioning as a histone deacetylase inhibitor that specifically targets both class I and class IIb HDAC. It received approval from the National Medical Products Administration (NMPA) of China in 2014 for the treatment of peripheral T-cell lymphoma (PTCL) and in 2019 for use in postmenopausal patients with advanced breast cancer, in combination with exemestane, a steroid-based aromatase inhibitor [133,151].

Tucidinostat received approval in China following the findings from a phase 2 trial that examined the drug used alone in patients with relapsed or refractory PTCL. The overall response rate recorded was 29%, with a complete response rate of 14%. The median period of progression-free survival was 2 months, while the overall survival duration was 21 months. The adverse events related to the treatment mainly consisted of blood-related issues, including thrombocytopenia at 51%, leukopenia at 40%, and neutropenia at 22% [133].

The combination of tucidinostat and exemestane, which is used to treat breast cancer, is also included in the targeted treatment for solid tumors. A Phase III study was conducted on tucidinostat and exemestane for postmenopausal women with advanced, hormone receptor (HR)-positive breast cancer (ACE). There were 365 participants, with 244 from the tucidinostat cohort and 121 from the placebo. The median progression-free survival (PFS) was 7.4 months in the tucidinostat group, while the exemestane monotherapy group had a median PFS of 3.8 months [152].

The Tucidinostat is currently approved in China but has not yet received approval from the U.S. Food and Drug Administration or European Medicines Agency (EMA) for use in either hematologic or solid tumours [133].

Four HDAC inhibitors have received approval from the US FDA for the treatment of specific types of hematological malignancies. While their effectiveness against these blood cancers is noteworthy, monotherapy with HDAC inhibitors has not proven to be sufficient for treating solid tumors.

#### 3.1.2. BET Inhibitors 

The latest research has concentrated on developing strategies that specifically target individual BET proteins or individual bromodomains to diminish the toxicity associated with pan-BET protein inhibition. Primarily, this approach has also been proven to yield favorable effects in cancer treatment. Selective inhibitors for BET-BD1 and BET-BD2 have been shown to represent more effective therapeutic strategies for cancer and inflammatory diseases, respectively, as the toxicity linked to the inhibition of a single BET-BD appears to be substantially lower than that associated with pan-BET-BD inhibition [153]. Nonetheless, the distinct application of BET-BD1 and -BD2 inhibitors in cancer and inflammatory diseases requires further refinement through more comprehensive investigation into their mechanisms of action. Bromodomain inhibitors that possess the ability to modulate BET protein function to regulate gene transcription selectively likely hold substantial potential as new epigenetic therapies for a diverse range of human diseases, such as cancer [154].

##### Small-Molecule BET Inhibitors

The synthesis of the first two small-molecule BETi in 2010 initiated more thorough research on more BET inhibitors. These two inhibitors were JQ1 and I-BET762(GSK525762A, molibresib, I-BET) [155].

(A) JQ1 (thieno-triazole-1,4-diazepine)

JQ1 is a small molecule thienotriazolodiazepine-based, selective BETi. Out of all the bromodomain proteins in the human body, it binds most effectively to BRD4. JQ1 has a strong antiproliferative effect against BRD4-dependent cancer cell lines and NUT midline carcinoma (NMC) [156]. The inhibitor mimics acetylated lysine and competitively binds to BD1 and BD2 via hydrogen bonding with an asparagine residue in the binding pocket [157,158]. 

Many tumor models, including medulloblastoma, breast and lung cancer, show an anti-tumor response to JQ1. Although it has an anti-tumor impact, JQ1 has a suboptimal pharmacokinetic profile, little oral bioavailability, and a half-life of 1 h, so the drug must be given twice daily most of the time to obtain the intended result [155,159].

(B) JQ2

Forthwith, a synthesis of a promising analogue of JQ1, TEN-010 (also known as JQ2), has been made. TEN-010 has shown preliminary improved pharmacokinetic properties, including a longer half-life, but it is still undergoing clinical trials in patients with AML, myelodysplastic syndrome, and other solid tumors [155,157,160].

(C) I-BET762

The second small-molecule inhibitor developed in 2010, by GlaxoSmithKline, is benzodiazepine I-BET762. Its advantage over JQ1 is that it can be administered orally. I-BET762 inhibits the bromodomains of BRD2, BRD3, and BRD4. It was initially designed to reduce inflammation by separating the BET proteins from the enhancer region of activated macrophage inflammatory genes [161]. It has been tested against numerous cancer models, and it has shown effectiveness, the same as JQ1, in fighting MM models in vivo [162].

This inhibitor has been shown to have potent anti-cancer activity in numerous preclinical models, including pancreatic cancer and neuroblastoma [163,164]. It has recently been studied in clinical trials for NMC, small-cell lung cancer, castration-resistant prostate cancer, triple-negative breast cancer, and gastrointestinal stromal cancer. However, many adverse events were observed [157].

(D) OTX015

Another small molecule BETi, OTX015, is also structurally similar to JQ1, but with better oral bioavailability [165]. The same as I-BET762, it inhibits BRD2, BR3, and BRD4 bromodomain-containing proteins. This inhibitor has shown positive effects on fighting B-cell lymphoma [159], MM [166], and in some solid tumors, including neuroblastoma, mesothelioma, and sarcoma [167].

The clinical advantage of most BETi evaluated to date has been limited due to unexpected toxicities. In one of the first clinical trials testing OTX015 against hematopoietic and solid cancers, the patients showed multiple toxicities caused by the dose of the drug, such as gastrointestinal disorders, anemia, thrombocytopenia, hyperbilirubinemia, fatigue, headache, and back pain [168,169]. Latterly, a clinical trial using a new BETi, BAY1238097, against solid cancers has been brought to an end, because of dose-limiting toxicities [155,170]. Findings of these many toxicities connected with the use of small molecule BETi suggest that earlier generations of BET inhibitors share a common toxicity profile [157,171,172].

##### Selective BETi (BD1/BD2)

Older generation pan-BET inhibitors bind with the same affinity to both bromodomains, BD1 and BD2. It was found that to eliminate BETi-related toxicity, inhibitors should target only one of the bromodomains of BET proteins [173]. Selective BD2 inhibitors have been shown to have potent tumor-suppressive properties, which is why most selective BETi have been developed against BD2, but studies report that targeting only BD1 is also sufficient to induce the desired anti-cancer effect [153]. However, it has been shown that BD2 is more critical for activating interferon-response genes [171]. A recent study compared a panel of 18 BET inhibitors for their selectivity towards BD1 and BD2. This study showed that only ABBV-744, iBET-1, and iBET-2 showed selectivity, with the new “iBET” molecules showing the highest selectivity reported to date. Interestingly, targeting only BD1 was found to be sufficient to phenocopy the antiproliferative effects of the pan-BETi in vitro. iBET-1 also showed potent antitumor properties in vivo [157,174]. Many BETi selective for one of the bromodomains have been developed to date. Among others, we can distinguish two new compounds: iBET-BD1 (GSK778), which shows selectivity for BD1, and iBET-BD2 (GSK046), selective for BD2 [153,157].

(A) ABBV-744

A newly discovered BD2-selective BET protein inhibitor is ABBV-744. It is highly selective against BD2 of BRD2, BRD3, and BRD4 and has strong antiproliferative activity against AML and prostate cancer cell lines, with a very low half maximal inhibitory concentration (IC50). Beneficial activity has also been demonstrated in prostate cancer xenografts in vivo, which responded to a dose as low as 4.7 mg/kg; this is associated with the low toxicity of ABBV-744. Compared to the doses in studies using JQ1 (50–100 mg/kg was administered in vivo), it is a significant improvement [155,175].

(B) SJ432

Another effective BET inhibitor, selective for the BD2 domain, is SJ432. It is much more potent than JQ1 in vivo against neuroblastoma. SJ432 shows strong antitumor activity at a low effective dose of 15 mg/kg, which, although higher than that of ABBV-744 (4.7 mg/kg), still maintains potent efficacy while avoiding the high-dose toxicity commonly observed with other BET inhibitors [86,155]. Both ABBV-744 and SJ432 show significant improvement compared to JQ1, the prototype of pan-BET inhibitors, which had antitumor activity in vivo at doses of 50–100 mg/kg and showed greater toxicity than the new BETi. The results of studies on these two inhibitors, selective for the BD2 bromodomain, are divergent from the position of Gilan et al. that inhibition of only the BD1 domain is necessary to induce antitumor effects [153]. This suggests that BD1 and BD2 bromodomains have different functions in the development of different types of cancers [157]. 

Additionally, a new study on radiation-induced profibrotic fibroblast response shows that BD2-selective inhibitors may effectively prevent radiation-induced fibrosis. Previously mentioned GSK046 inhibits radiation-induced profibrotic marker genes without significant toxicity [157,174].

##### Bivalent BET Inhibitors

The above-described selective BET inhibitors (BD1/BD2), despite the ability to specifically bind a single bromodomain, still retain the properties of pan-BET inhibitors. They are not able to distinguish between different BET proteins within their group, due to the structural similarity of the protein family (Brd2, Brd3, Brd4, and Brdt) [153,175,176,177].

Bivalent BET inhibitors enable specific targeting against a single BET family protein. Bivalent BETi (biBET) bind two BRDs simultaneously, which leads to slower dissociation kinetics and increased cell potential. Some of the first reported bivalent BET inhibitors (MT1 and biBET) exhibit potent BRD4 binding and inhibition properties compared to their monovalent counterparts [178,179].

A recently discovered bivalent inhibitor, AZD5153, increases BRD4 displacement from chromatin at lower concentrations than I-BET762. It acts by simultaneously binding Brd4, BD1, and BD2. AZD5153 demonstrates potent antitumor activity in hematological and thyroid cancer models in vivo at doses of 5–10 mg/kg and GI50<25 nM [180,181]. New studies are emerging to develop oral bivalent BET inhibitors with short and hydrophilic side chains, as their predecessors with longer linker chains have shown limited metabolic stability by liver enzymes [157,182]. Older BET inhibitors cannot distinguish BRDT from BRD4. Although BRD4 is a promising anticancer target, inhibition of BRD4 induces simultaneous inhibition of BRDT, which is an undesirable effect. Targeting BRDT is used in the design of non-hormonal male contraception because it is expressed only in the testes [176,183,184]. Therefore, inhibitors selective for BRDT were needed. Guan et al. created a new bivalent inhibitor capable of stabilizing different conformational states of BRDT and BRD4, which has greater selectivity for BRDT [157], by modifying a non-selective monovalent BET inhibitor [185]. 

##### BRD4-Selective Inhibitors 

Brd4-selective inhibitors may be more beneficial than pan-BET inhibitors [186].

(A) AZD5153

Already mentioned, AZD5153 is a BRD4-selective BETi that is extremely effective in displacing BRD4 from chromatin. It owes its properties to its simultaneous binding to both bromodomains (BD1 and BD2) of Brd4 [176,187]. In preclinical models, AZD5153 has demonstrated potent antiproliferative and apoptosis-inducing properties in many cancer cells, including colon cancer. It has also been found to inhibit Wee1 kinase expression and negatively affect the cell cycle checkpoint at the G2/M level. It also enhances the antitumor activity of the PARP inhibitor in vivo and in vitro [157,188].

(B) NHWD-870

NHWD-870 is a new selective inhibitor for the Brd4 protein. It differs in its core structure from the older generation BETi (JQ1, OTX-015, I-BET762, and CPI-0610). In addition, it is expected to be more potent than the BET inhibitors currently in clinical development (BMS-986158, OTX-015, and GSK-525762). NHWD-870 has many beneficial properties, including inhibition of tumor proliferation by inhibiting BRD4 and c-MYC transcription, inhibition of tumor-associated macrophages (TAM) development, and reduction of CSF-1 (colony-stimulating factor) secretion by tumor cells [189,190].

(C) ZL0513

ZL0513 is another potent, selective BRD4 inhibitor. Studies show that it has antiangiogenic effects in models of the chorioallantoic membrane and the yolk sac membrane of the chick embryo. Antiangiogenic effects are desirable in anticancer therapies because the process of angiogenesis facilitates the development of cancer tumors by providing them with oxygen and nutrients via newly formed vessels. The BRD4 protein drives angiogenesis by releasing the pause of RNA polymerase II (RNAPII) [191]. Therefore, potential inhibitors selective for Brd4, also acting antiangiogenically, may be desirable in cancer therapy [192].

Recent research indicates that inhibiting BET influences DNA repair processes by heightening replication stress and disrupting homologous recombination (HR). BET inhibitors cause replication forks to stall and result in DNA damage, which leads to an increase in RAD51, an important protein involved in homologous recombination, at the sites of DNA damage. Nonetheless, this HR engagement is frequently inadequate or flawed, making cells more responsive to PARP inhibitors and therapies that cause DNA damage. This indicates that BET inhibitors influence DNA repair by raising replication stress and hindering effective HR-mediated repair. This provides a reason for using combination therapies in cancers that have faulty DNA repair mechanisms [193].

#### 3.1.3. KDM4 Histone Demethylase Inhibitors

In healthy cells, histone lysine demethylases (KDMs) participate in maintaining homeostasis; deregulation of these enzymes is characteristic of cancer cells. For this reason, they have become a new therapeutic target in cancer treatment, and some KDM inhibitors have shown antiproliferative activity [194]. KDMs are classified into eight subfamilies, known as KDM1 through KDM8, according to their substrate specificity, conserved domains, and enzymatic mechanisms [42]. The KDM4 subfamily of H3K9 histone demethylases plays a vital role as epigenetic regulators. They influence chromatin structure and gene expression by demethylating histones H3K9, H3K36, and H1.4K26. The KDM4 subfamily comprises four main proteins—KDM4A, KDM4B, KDM4C, and KDM4D—each featuring the JmjC domain but exhibiting distinct substrate specificities. KDM4 proteins are found to be overexpressed or deregulated in various cancers, including breast, colorectal, pancreatic, lymphoma, and gastric cancers, cardiovascular diseases, and cognitive impairments, making them promising targets for therapeutic intervention. The KDM4A-C enzymes contain a catalytic histone demethylase domain, which includes both JmjN and JmjC domains and non-catalytic double prolyl hydroxylase (PHD) and Tudor domains. In contrast, KDM4D possesses only the catalytic domain and does not feature the PHD and Tudor domains [194,195]. 

Scientists currently categorize the available KDM4 inhibitors into four main types:

1. 2-oxoglutarate (2-OG) cofactor mimics;
2. Metal cofactor disruptors;
3. Histone substrate competitive inhibitors;
4. Substrate- and cofactor-independent inhibitors [194,196].

We will summarize the current knowledge on KDM4 inhibitors, focusing primarily on their mechanisms.

The most numerous KDM4 inhibitors are 2-oxoglutarate cofactor inhibitors. These inhibitors act by chelating Fe (II) in the JmjC catalytic domain, thereby preventing α-ketoglutarate (α-KG) binding and blocking histone demethylation [197]. 2-OG is essential for the catalytic activity of all JmjC-containing demethylases. Therefore, one strategy for inhibition is the development of α-KG analogs, which can either compete with α-KG for binding or act as metal chelators. While some 2-OG analogs are potent inhibitors, their large structures limit cellular permeability [194]. Among 2-oxoglutarate analogs, compounds such as N-oxalylglycine (NOG), pyridinedicarboxylic acid (PCA), and 8-hydroxyquinoline (8-HQ) act as cofactor mimics that inhibit KDM4 enzymes by chelating Fe(II) in their catalytic site, thereby blocking demethylase activity. These compounds are described as KDM4 inhibitors and have demonstrated antiproliferative activity in vitro, with IC_50_ values of 78 µM (NOG), 1.4 µM (PCA), and 0.6 µM (8-HQ), although none have been approved for clinical use in cancer treatment [198].

The latest study has shown the effect of TACH101, which is an innovative, first-in-class small-molecule inhibitor targeting the epigenetic regulatory enzyme KDM4. Remarkably, this compound demonstrates strong inhibitory effects on all four KDM4 isoforms (A-D). Additionally, TACH101 elevates levels of H3K36me3 and triggers apoptosis in human esophageal cancer, triple-negative breast cancer, and colorectal cancer cell lines. A Phase 1 clinical trial evaluating TACH101 in patients with advanced and metastatic tumors has been initiated and is currently underway. Despite the positive prognosis, TACH101, like NOG and PCA, has not yet received official approval for cancer treatment [199].

Metal disruptors can be classified into nonferrous metals or organic molecules. These disruptors inhibit histone demethylation by interfering with the binding of Fe^2+^ ions in the active site of KDM4 enzymes, which contains a Zn–Cys_3_–His coordination. While this category of inhibitors exhibits rapid reaction kinetics, the binding site is located on histones, and numerous sites on other proteins possess a similar structural configuration [200]. A representative of nonferrous metals is nickel (Ni). KDM4A and KDM4C are inhibited by Ni (II) ions. Through the use of X-ray Absorption Spectroscopy (XAS), it has been shown that the inhibitory effect of nickel on these enzymes stems from its occupancy in the active site in place of iron. Importantly, findings indicate that the presence of nickel does not interfere with the binding of the αKG cofactor in the active site [201]. An example of an organic molecule is 2-(1H-tetrazol-5-yl)acetohydrazide, which is recognized as a selective inhibitor of KDM4A. This inhibitor has a low relative molecular mass and low complexity and demonstrates a high affinity for the KDM4A metal-binding site, with an inhibitory concentration (IC50) value of 46.6 μM as determined by an FDH-coupled assay, and a significantly lower IC50 value of 2.4 μM as measured through antibody-based analysis. This molecule exhibits a higher specificity for KDM4A compared to other KDMs, making it an ideal candidate for targeted inhibition [202]. 

While metal disruptors exhibit varying degrees of specificity, organic chelators tend to offer higher selectivity, making them more promising candidates for therapeutic use. Further research is essential to optimize their specificity and evaluate their potential applications in cancer treatment and other diseases [200].

Histone substrate competitive inhibitors include JIB-04 and NSC636819. All these inhibitors have anti-cancer activity but have not been approved for clinical use by the FDA and are still in the research phase. The inhibitor JIB-04, which is specific to the Jumonji family, effectively blocks the catalytic activity of KDM4A, KDM4B, and KDM4E both in vitro and in vivo [203]. The KDM4-targeting JIB-04 epigenetically activates the natural innate immune response of the tumor, causing immunogenic cell death (ICD), and then leading to marked antitumor outcomes [204]. Some studies have shown its therapeutic potential in the treatment of HCC malignancy and colorectal cancer. However, these are studies at an early stage [205]. The potential of using NSC636819 in the treatment of prostate cancer has also been demonstrated because the viability of cultured prostate cancer cells is significantly reduced due to NSC636819 activity, and the expression of growth-related genes is also inhibited [206].

The last group of KDM4 inhibitors is substrate- and cofactor-independent inhibitors. Cyclic peptides and non-catalytic domains targeting KDM4 inhibitors belong to them. Cyclic peptides attach to allosteric sites and less conserved regions of KDM4C found on the surface, distant from the active site. These initial findings could eventually aid in discovering new binding sites for KDM4C-selective inhibitors [203]. Two cyclic peptides aimed at the histone demethylase KDM4C were discovered and optimized as inhibitors through amino acid substitution, truncation, and chemical alterations [204]. This group of KDM4i also includes non-catalytic domain targeting inhibitors. PHD fingers, present in JmjC-KDM enzymes like KDM2s, KDM4A-C, KDM5s, and KDM7s, facilitate histone binding. Recent studies have identified small-molecule inhibitors targeting PHD finger domains in certain KDM family members; however, inhibitors for non-catalytic domains, such as the PHD fingers of KDM4, remain undiscovered [196].

#### 3.1.4. DNMT Inhibitors 

Disturbed DNA methylation in cancer cells can be considered in three aspects: (1) DNA methyltransferase is overexpressed, and its catalytic activity is increased in tumor cells; (2) DNA methylation in repetitive sequences of tumor cells is significantly decreased, which leads to chromosomal instability; (3) The tumor supressor genes are inhibited due to hypermethylation in its promoter [207,208]. Currently, five types of DNMT have been distinguished, among which DNMT1 and DNMT3A/3B are related to DNA methylation [12,209]. A lack of DNA methylation is connected with the promotion of proto-oncogenes. Hypermethylation is associated with the silencing of TSG [208,210].

The DNMT inhibitors have a great impact on tumor immunotherapy. These inhibitors promote tumor antigen presentation by increasing the expression of tumor-associated antigen (TAA). At the same time, they enhance the cytotoxic T lymphocytes (CTL) by upregulating the expression of IL-2, IFN-γ, and other cytokines through demethylation. The DNMTi have an impact on NK cell-mediated cytotoxic effect, and can remodel the chromatin of tumor cells by changing DNA and histone methylation [208]. Additionally, DNMT inhibitors enhance immunoreactivity. For instance, DNMT inhibitors can upregulate the major histocompatibility complex I (MHC-I) by obstructing DNA hypermethylation in the promoter region of MHC-I [211]. DNMT inhibitors furthermore augment the cytotoxic effects of CD8^+^ T cells and assist CD4^+^ T cells and NK cells by promoting the expression of cytokines such as IL-17 [208,212]. DNA methyltransferase inhibitors, including 5-azacytidine and decitabine, have demonstrated the ability to counteract the hypermethylation of tumor suppressor genes, thus reinstating their expression. Significantly, this process of demethylation can also restore the activity of suppressed DNA repair genes, thereby improving the DNA damage response in cancer cells. For instance, it has been noted that DNMT inhibitors can reactivate the expression of the DNA repair gene MLH1, which is frequently turned off due to promoter hypermethylation in several types of cancer. This reactivation enhances the ability to correct mismatches in DNA and makes cancer cells more responsive to agents that cause DNA damage. Furthermore, inhibiting DNMT may result in alterations in chromatin accessibility, which can help attract DNA repair proteins to areas of damage. This could enhance genomic stability and the effectiveness of chemotherapy in cases with impaired DNA repair [1,2,51,213]. 

Four DNMT inhibitors: Azacitidine, decitabine, clofarabine, and arsenic trioxide have been approved by the United States FDA for the therapy of myelodysplastic syndrome and hematological diseases. 

##### DNA Methyltransferase Inhibitors Available for Patients

(A) 5-Azacitidine

5-Azacitidine undergoes modification by nucleotide reductase and is subsequently phosphorylated by uridine cytidine kinase to create a triphosphate, 5-azacitidine triphosphate, which is incorporated into the DNA as a cytosine analogue during DNA replication, thereby hindering DNA cytosine methylation [214]. 5-Azacitidine is a Cytarabine derivative, and it was approved for the management of MDS in 2004 under the brand name Vidaza [215]. It may also impede tumor growth in intracerebral glioblastoma models in vivo [208]. The drug’s overall response (OR) was 13.9% in treating patients with chronic myelomonocytic leukemia (CMML) and acute myeloid leukemia (AML). Significant adverse reactions, particularly thrombocytopenia, febrile neutropenia, and elevated temperature, were recorded [208].

(B) Decitabine

Deoxycytidine derivative, known as Decitabin, received approval for the management of MDS under the commercial name of Dacogen. It is converted to a 5-azabine triphosphate by deoxycytidine kinase without any preceding deoxidation. The inhibitory efficacy of DNMT is thirty times greater than that of azacitidine [208,214]. In the treatment of MDS, Decitabine has been shown to have a complete response (CR) of 20% and an overall response (OR of 61%; the overall survival (OS) was 20 months. The primary adverse effects include a decrease in neutrophils, feelings of nausea, and tiredness [208].

(C) Clofarabine

Clofarabine is a purine nucleoside DNMT inhibitor, utilized in refractory or relapsed AML in pediatric patients under the brand name clofarabine. Clofarabine blocks the proliferation of tumor cells by decreasing DNMT1 and inhibiting the methylation of TSGs, such as PTEN, antigen-presenting cells (APC), and RARβ2 [216]. Clofarabine has been used in the treatment of patients with relapsed AML or elderly patients with newly diagnosed AML; the OR was 43%. The frequently occurring negative effects included low platelet levels and a shortage of red blood cells [208].

(D) Arsenic trioxide

Arsenic trioxide has been approved for the treatment of relapsed or persistent acute promyelocytic leukemia (APL) under the trade name Trisenox [217]. This medication facilitates the apoptosis of leukemia cells by hindering DNA methylation [218]. During the treatment of patients with APL, all of them achieved hematologic complete remission. Adverse effects included unusual blood clotting and low red blood cell count [208]. 

##### DNA Methyltransferase Inhibitors Undergoing Clinical Trials

(A) Guadecitabine

Guadecitabine (SGI-110) is a dinucleotide derivative of decitabine, which inhibits DNA methylation like decitabine, but is more stable due to the phosphodiester bond of SGI-110. SGI-110 is utilized for the therapeutic intervention of MDS and AML in clinical settings. The most frequent grade 3 side effects included febrile neutropenia, pneumonia, and low platelet count [208].

(B) RX-3117

RX-3117 may reduce the growth of breast, lung, and colon cancer cells in vitro. The negative consequences included a lack of red blood cells, tiredness, and loose stools [219].

(C) 5-Fluoro-2′-deoxycytidine

The pyrimidine analog, 5-fluoro-2′-deoxycytidine (FdCyd), was developed by adding a fluorine atom to the pyrimidine ring to increase the compound’s metabolic stability and enhance its ability to inhibit DNA methylation [219].

(D) Fazarabine

Fazarabine is an Ara-C and azacitidine derivative. Its cytotoxicity against the Molt-4 human lymphoblastic leukemia cell in vitro has been established; however, the studies were discontinued due to an unsatisfactory response [220].

(E) Cladribine and fludarabine

Adenosine analogs DNMTi cladribine and fludarabine are applied for the treatment of hematologic tumors in clinics [208].

(F) Procaine

Procaine (10 nM) impeded the proliferation of MCF-7 breast cancer cells and diminished the methylation of the tumor suppressor gene RARβ [208,221].

(G) Epigallocatechin gallate

Epigallocatechin gallate (EGCG, 13) is derived from green tea. EGCG inhibited the methylation of WIF-1 in lung cancer cells [222] and GSTP1 in prostate cancer cells [208].

(H) Hydralazine

Hydralazine is a muscle relaxant administered in the treatment of hypertension. It decreases DNA methylation in systemic lupus erythematosus cells. Hydralazine prompted demethylation and increased expression of tumor suppressor genes p16 and RAR2 in human bladder cancer and APC in human cervical cancer HeLa and Caski cells [223].

(I) Genistein and equol

Genistein and equol are flavonoid composites. Equol prompted the promoter hypomethylation of tumor suppressor genes BRCA1 and BRCA2 in breast cancer cells [224]. Equol reduces the risk of prostate cancer in men and breast cancer in women. Genistein, in isolation, had adverse effects that included headaches, sickness, and unexpected warmth [208].

(J) Curcuminum

A diketone derivative, curcumin, possesses anti-inflammatory, anti-tumor, anti-angiogenesis, anti-bacterial, and immunomodulatory properties. Curcumin obstructs the DNA methylation of RAR2 and FANCF in cervical cancer cells in vitro. Symptoms included facial swelling, decreased blood volume, and reduced red blood cell count [225].

(K) Disulfiram

Disulfiram is an alcohol abuse drug, but it may inhibit DMNT1 and stimulate the expression of tumor suppressor genes, such as p16 and RARβ2. Disulfiram is presently undergoing phase II clinical trials for the treatment of prostate cancer [208,225].

(L) Resveratrol

Resveratrol is extracted from peanuts with a cardiovascular protection effect. It exhibits synergistic effects when used alongside cladribine or fludarabine. It lowers the expression of DNMT1 [226].

(M) Caffeic acid

Caffeic acid, which is a phenolic acid, prevents DNA methylation in a non-competitive manner [208]. 

Since 2004, the initial nucleosides DNMTi, 5-azacitidine, clofarabine, and decitabine have been approved for the treatment of MDS, CMML, and AML. Nevertheless, the composition of 5-azacitidine is highly unstable. To enhance the drug’s stability, second-generation cytidine analogs, such as Zebularine, have been developed [208]. 

#### 3.1.5. Nicotinamide N-Methyltransferase (NNMT) Inhibitors

NNMT is an enzyme found in the cytoplasm that is essential for maintaining the balance of energy in cells by controlling the level of (nicotinamide) NAM and S-adenosyl-L-methionine (SAM) within the important pathways of NAD+ recycling and the methionine cycle. Recent investigations into NNMT mainly focus on cancer, as it has been discovered to have increased levels in various forms of solid tumors [227]. Cancer cells that express NNMT have a changed epigenetic condition that features low levels of methylation on histones and various other proteins linked to cancer. Targeting NNMT might prove to be a promising therapeutic strategy to influence the cancer epigenome for treatment advantages. The use of potent, specific, and cell-active inhibitors of NNMT is essential for investigating its regulatory functions and exploring alternative pharmacological theories that suggest it as a treatment target [228].

(A) GYZ-319, a bisubstrate NNMT inhibitor with an IC_50_ of 3.7 nM, displays minimal cellular activity due to poor membrane permeability linked to its polar amine and carboxylic acid groups. To address this, ester-based prodrugs such as TML-GYZ-319-isopropyl ester were synthesized to mask these functionalities. The prodrug showed enhanced cellular uptake and restored NNMT inhibition in vitro. These findings support the use of prodrug strategies to optimize the pharmacological profile of potent but cell-impermeable NNMT inhibitors [229].

(B) A novel class of bisubstrate NNMT inhibitors has been developed, incorporating non-benzamide nicotinamide mimetics and a trans-alkene linker architecture. The lead compound, 17u, a para-cyano-substituted styrene derivative, demonstrated high biochemical potency (IC_50_ = 3.7 nM) and strong target affinity (KD = 21 nM). Despite its potent enzymatic inhibition, compound 17u exhibited limited cellular efficacy, attributable to poor membrane permeability. These findings provide a new structural framework for the design of next-generation NNMT inhibitors with therapeutic relevance [230].

(C) A novel class of nicotinamide N-methyltransferase (NNMT) inhibitors are macrocyclic peptides. Out of 17 candidates, peptides 4, 6, 13, 15, and 17 showed potent inhibition (IC_50_: 229–772 nM). Peptide 17 emerged as the most potent, demonstrating an IC_50_ of 229 nM. Peptides 4 and 13 significantly reduce MNA levels in both A549 lung carcinoma cells and human aortic endothelial cells (HAEC). Their effect was comparable to the small-molecule inhibitor 6-MANA, underscoring their cellular activity and therapeutic potential as allosteric NNMT inhibitors [231].

Several NNMT inhibitors have been developed and show substantial potential as therapeutic strategies to address chemoresistance; however, to date, none have been approved for cancer treatment by the FDA. An overview of clinically approved and investigational epigenetic inhibitors is provided in Table 2.

Several studies indicate that epigenetic inhibitors can exhibit promising specificity by preferentially reactivating silenced genes in cancer cells [232]. In cancer cells, DNMT inhibitors showed high target selectivity, predominantly upregulating genes silenced by promoter DNA methylation, with minimal effects on gene downregulation. In contrast, HDAC inhibitors exhibited much lower selectivity, affecting about one-third of the transcriptome by both up- and downregulating gene expression, including many genes not altered in cancer. The lack of selectivity may help explain the numerous adverse effects experienced by patients treated with HDAC inhibitors [233]. Concerns remain about off-target activity and toxicity in normal proliferative tissues, underscoring the need for refined targeting strategies and improved therapeutic approaches.

### 3.2. Combination Therapies with DNA-Damaging Chemotherapeutics

Resistance to monotherapy, including chemotherapy and radiotherapy, is a common challenge faced by cancer patients, highlighting the need for continuous research and the development of more effective treatment strategies, such as combination therapies with DNA-damaging agents [234]. Combination therapies combine at least 2 different therapeutic agents and are used to improve the effectiveness and reduce resistance to monotherapy. For this reason, they are widely used in cancer treatment [235]. Various forms of combined therapies have been developed. Some have already received approval from the U.S. Food and Drug Administration, while others have not due to limited effectiveness or adverse effects

One example of combination therapies is the combination of traditional forms of monotherapy, such as chemotherapy and radiotherapy, with epigenetic drugs. The primary focus will be directed towards this therapy. HDAC inhibitors and DNMT inhibitors enhance overall chromatin relaxation, making it easier for genotoxic agents to cause DNA damage and disrupting the processes involved in DNA damage repair. Therefore, the latest research focuses on the use of these inhibitors [236]. Scientific work on the effectiveness of using these epigenetic drugs with chemotherapeutics is still ongoing. Cisplatin exerts its cytotoxic effects primarily through the formation of intra- and interstrand DNA crosslinks, which interfere with DNA replication and transcription, ultimately leading to the formation of double-strand breaks during the repair process [237]. The potential activity in combination of decitabine (DNMT inhibitor) with cisplatin (chemotherapeutic) in the treatment of various solid tumors remains under investigation. One study found that combination therapy with cisplatin and decitabine significantly decreases HNSCC (head and neck squamous cell carcinoma) growth and HNSCC pain [238]. Other studies have found that the combination of decitabine and cisplatin showed moderate activity in advanced cervical squamous cell carcinoma patients, especially in those with metastatic disease at sites not previously irradiated [239]. Additionally, another study demonstrated that the combined use of cisplatin and decitabine can promote demethylation of the Sox2 gene promoter region, resulting in increased Sox2 expression. This upregulation was linked to anti-tumor activity in gastric cancer cells, possibly through the reactivation of tumor-suppressive pathways regulated by Sox2 [240]. 

All these studies lead to the conclusion that DNMT inhibitors may constitute the basis of combination therapies in the treatment of solid tumors. Furthermore, many preclinical studies have shown the notable radiosensitization effect of DNMTis. The clinical trials, such as NCT03445858, NCT01707004, and NCT04174196 that are currently recruiting or have concluded, concentrating on lymphoma and hematological neoplasms, have indicated the potential of DNMTis in conjunction with RT [241]. Researchers are also focusing on the use of other epigenetic drugs, including HDAC inhibitors. A phase II study in NSCLC patients found that combination therapy of Vorinostat (HDAC inhibitor), carboplatin, and paclitaxel improved survival outcomes compared to a placebo, with a 1-year survival rate of 51% versus 33%. However, Vorinostat increased toxicity, and the phase III trial was halted due to limited effectiveness. This resulted in the US FDA not approving this combination therapy [241]. 

The FDA has not approved any combination therapies that combine epigenetic drugs with radiation therapy for the treatment of cancer. Researchers are focusing on the possibility of using HDACi, which is related to increasing the radiosensitivity of cells. One of the examples of HDACi used is Panobinostat. In a recent study, the authors demonstrated that low concentrations of Panobinostat, an HDAC inhibitor, when used in combination with radiotherapy, enhanced apoptosis in prostate cancer. Also, the combination treatment led to increased DNA damage and resulted in reduced activation of both the NHEJ and HR repair pathways [242]. 

### 3.3. Strategies for Overcoming Resistance to Epigenetic Therapies

Combination therapies of epigenetic drugs with other treatments, including targeted therapy, chemotherapy, and immunotherapy, offer a way to address the limitations (including resistance) of single-agent epigenetic therapies. Currently, the majority of combination therapies are in the clinical development phase [243]. 

Combining epigenetic drugs with targeted therapies prevents the reprogramming of kinases, which counters BCL-2 gene resistance and hormonal resistance. Combination therapies of epigenetic drugs with chemotherapeutics eliminate the epigenetic reprogramming associated with chemotherapy resistance.

The integration of epigenetic therapies with immunotherapy results in increased dsRNA expression, which is followed by an elevated number of APCs, thereby inducing IFN signaling, activating MHC class I molecules, and T lymphocytes. The outcome of these actions is the inhibition of drug resistance [244].

#### 3.3.1. Combining Epigenetic Drugs with Chemotherapy

The combination of epigenetic agents with chemotherapy drugs that cause DNA damage constitutes a promising approach to overcoming resistance to cancer treatment. Chemoresistance frequently entails alterations in DNA and histone modifications, which are subject to reversal through the application of specific inhibitors. Aberrant DNA methylation exerts an influence on critical genes involved in pathways that foster resistance in diverse tumors by modifying cellular behaviors such as survival and proliferation. For instance, the DNMT inhibitor decitabine can facilitate the re-sensitization of chemotherapy-resistant lymphoma cells to doxorubicin without significant adverse effects [245,246]. A clinical trial involving patients with diffuse large B-cell lymphoma (DLBCL) indicated that the administration of azacitidine followed by the conventional R-CHOP regimen was well-tolerated and resulted in a substantial rate of complete remission. Likewise, HDAC inhibitors can render cancer cells more susceptible to chemotherapy, with panobinostat demonstrating efficacy in diminishing resistance to cisplatin in lung cancer cells by influencing HIF-1α. This protein is also associated with resistance to a variety of other chemotherapy agents [244].

#### 3.3.2. Combining Epigenetic Drugs with Targeted Therapies

Targeted therapies have significantly changed cancer treatment by using chemicals designed to interact with specific mutant proteins. These therapies often show quick results in genetically defined groups, but resistance to these treatments is a common issue. Resistance can happen through genetic changes or changes in how genes are expressed, but these may be reversible with epigenetic therapies. In NSCLC, the role of epigenetic changes in resisting epidermal growth factor receptor tyrosine kinase inhibitors (EGFR-TKI) is still not fully understood. However, it has been suggested that drugs that inhibit HDAC, like MPT0E028, can help overcome this resistance by enhancing cell death when combined with first-line treatments like erlotinib, a frequently used epidermal growth factor receptor (EGFR) tyrosine kinase inhibitor used to treat non-small cell lung cancer. Studies have also shown that combining EGFR-TKIs with vorinostat can reverse resistance and promote cell death [247,248]. HDAC inhibitors can also combat resistance to mTOR inhibitors, as changes in gene methylation affect mTOR signaling. Several HDAC inhibitors have worked well with mTOR inhibitors in different cancers, showing potential for reversing resistance [244]. 

In blood cancers, DNMT inhibitors are being studied together with venetoclax, which works by inhibiting the anti-neoplastic protein BCL-2 that helps cancer cells survive. While venetoclax alone has shown promise in treating leukemia, resistance can develop quickly due to the upregulation of other anti-neoplastic proteins such as MCL-1 and BCL-XL [249,250]. When paired with azacitidine, DNMT inhibitors can lower MCL-1 levels and improve the effectiveness of treatment. This combination has shown a good safety profile and better outcomes for older patients with AML compared to using either drug alone. The FDA has recognized the combination of DNMT inhibitors and venetoclax as a “breakthrough therapy” for AML patients who cannot undergo intensive chemotherapy, and it is undergoing clinical trials for MDS and AML [251,252]. 

#### 3.3.3. Combining Epigenetic Drugs with Immunotherapy

By enhancing the body’s immune response against cancer cells, immune checkpoint therapies have made remarkable progress in the treatment of cancer [253,254,255]. These therapies employ immunoglobulins to inhibit checkpoint proteins such as CTLA-4, PD-L1, and PD-1, which can be effective but may encounter challenges such as inadequate antigen presentation and suboptimal T-cell responses. To address these concerns, researchers are investigating methods to utilize epigenetic remodeling. By inhibiting epigenetic regulators like LSD1, EZH2, HDAC, and DNMT, cancer cells can enhance the expression of certain molecules that facilitate the activation of immune responses, thus rendering them more amenable to immunotherapy [244]. 

Recent studies emphasize the potential of DNMT inhibitors to enhance the efficacy of immunotherapy across various cancer types. For example, decitabine has been demonstrated to augment T-cell activity in an ovarian cancer model and to improve survival rates when used in conjunction with anti-CTLA-4 antibodies [256]. Comparable effects have been reported in research related to prostate, colon, and breast cancers, where DNMT inhibition fostered T-cell infiltration and activity. Furthermore, a clinical trial focused on AML illustrated that the combination of azacitidine with a PD-1 antibody produced favorable outcomes in patient response and survival [244]. 

HDAC inhibitors have also indicated potential benefits when combined with immunotherapies, such as panobinostat, enhancing the effectiveness of anti-PD-1 therapy in melanoma, and belinostat improving anti-CTLA-4 treatment in liver cancer models [257]. Additional studies propose that entinostat can amplify the effects of IL-2 and vaccination in prostate and kidney cancers [258,259]. Current clinical trials are examining how the integration of epigenetic agents with immunotherapies may improve cancer treatment results. Future research will advance the understanding of the effects of these agents on both tumor and immune cells, leading to improved strategies for combination therapies [259]. 

#### 3.3.4. Using Nanotechnology to Target Epigenetic Drugs

Epigenetic medicine faces significant challenges despite progress in the field. The epigenetic drugs approved by the US FDA are not selective and often cause unwanted side effects, leading to toxicity and difficulties in achieving long-term treatment results. Key barriers to the broader use of these drugs include low permeability, solubility issues, and poor pharmacokinetic properties like low bioavailability and stability [260]. Improving drug delivery, stability, and specificity is crucial for unlocking their therapeutic potential. Approaches like nanoscale delivery systems and prodrugs could help by enabling targeted delivery to tumors and enhancing bioavailability while protecting against early degradation. Research is also exploring second-generation nucleoside analogues to improve tolerability and stability. Combining nanoparticles with epigenetic drugs and chemotherapy may provide better therapeutic outcomes with fewer side effects [261,262]. While many clinical trials show promise for using nanoparticle delivery systems to target siRNAs in cancer, more investigation is needed to determine the safety and efficacy of these delivery methods [263]. Non-coding RNAs, which comprise long non-coding RNAs (lncRNAs) and microRNAs (miRNAs), are crucial in managing DNA repair processes. For example, long non-coding RNAs (lncRNAs) such as DDSR1 and LINP1 assist in homologous recombination by attracting repair factors, whereas microRNAs (miRNAs) like miR-24 and miR-182 can reduce the expression of essential repair proteins like H2AX and BRCA1, thereby affecting both repair efficiency and the stability of the genome [264]. Focusing on these non-coding RNAs using nanocarriers presents a hopeful method to selectively influence DNA repair mechanisms in cancer cells.

Nevertheless, even with progress made, nanotherapeutic approaches encounter challenges, such as possible epigenetic changes induced by nanoparticles that could lead to cell damage or unintentional alterations in gene expression [265]. The variety of tumor microenvironments and the difficulties in achieving accurate targeting and regulated release also restrict clinical application. Current advancements in the design of nanoparticles, including stimuli-responsive and multifunctional nanocarriers, are being explored to address these challenges and enhance treatment results. In summary, combining nanotechnology with epigenetic treatment and targeting non-coding RNA shows great potential but needs additional refinement to guarantee safety and effectiveness [266]. 

#### 3.3.5. Resistance to BET Inhibitors

Small-molecule BET inhibitors used in clinical trials against glioblastoma (GBM) did not show the desired effects. One of the possible mechanisms responsible for this is the intrinsic resistance to BETi that develops during long-term treatment in clinical conditions. Jermakowicz et al. in their studies show that resistance to BET inhibitors appears within a few hours and involves the fibroblast growth factor receptor protein (FGFR1). Both inhibition of BET and FGFR1 proteins simultaneously act synergistically to reduce GBM tumor mass in vivo and in vitro. It can be concluded that simultaneous attack of BET and FGFR1 proteins may inhibit the mechanisms of intrinsic resistance and have a beneficial effect on inhibiting GBM cell proliferation in clinical settings [267].

Similarly, studies using BET protein inhibitors in treating AML have not shown satisfactory effects. Research by Shah et al. of the immediate epigenetic and transcriptional response after previous BET protein inhibition shows that BET inhibitor-mediated release of BRD4 from chromatin results in attenuation of the downregulation or may lead to an increase in the expression of specific transcriptional modules. This undesirable effect was found to be mediated by p300. How p300 regulates the development of BET inhibitor resistance differs between the different AML subtypes. Therefore, in some types of AML, p300 is most involved in the early stages of developing BET resistance, while in others, p300 is crucial at all stages of developing linear resistance to BET inhibition. The results of these studies suggest that simultaneous inhibition of BET and p300 proteins could prevent the development of resistance to BETi and thus improve their outcomes in the treatment of AML [268].

Other reported cases of BET inhibitor resistance include:

**(A)** Ovarian cancer, where long-term treatment with BET inhibitors causes reprogramming of the tyrosine kinase receptor, contributing to BET resistance [269]. 

**(B)** Activation of interleukin 6/8-Janus kinase 2 signaling induces BRD4 phosphorylation in colon cancer. Phosphorylation of BRD4 increases its stability and thus reduces the affinity with which BRD4 binds to BET inhibitors. This leads to the emergence of BETi resistance [270]. 

**(C)** In triple-negative breast cancer, resistance to BET protein inhibitors resulted from a bromodomain-independent mechanism [271]. 

Loss-of-function mutation of SPOP (BRD4 E3 ubiquitin ligase) in prostate cancer causes impaired ubiquitination-dependent BRD4 degradation, contributing to BETi resistance [272,273]. 

In conclusion, resistance to epigenetic therapies poses a serious challenge to their future development and application in cancer treatment. While there are strategies to overcome resistance, the topic remains largely unexplored and requires further research. 

## 4. Conclusions and Perspectives

Despite significant advances in understanding the role of epigenetic mechanisms in cancer, translating this knowledge into effective therapies remains challenging. One of the most significant barriers is tumor heterogeneity. The epigenetic landscape of cancer is highly variable, not only between different tumor types but also within a single tumor. This diversity, as described in our review, affects DNA methylation patterns, histone modifications, and chromatin remodeling, making it difficult to identify universal biomarkers and predict which patients will respond to specific epigenetic therapies. As a result, treatment efficacy can be inconsistent, and resistant cancer cell populations may quickly emerge.

Another critical issue is the reversibility and plasticity of epigenetic marks. While the dynamic and reversible nature of DNA methylation and histone modifications makes them attractive therapeutic targets, it also means that the effects of epigenetic drugs may be short-lived. Cancer cells can adapt by restoring aberrant epigenetic states or activating alternative pathways, which can lead to transient therapeutic effects or the development of resistance.

Off-target effects and toxicity in normal cells, particularly those with high proliferative potential, represent a further limitation of current epigenetic therapies. Agents such as DNA methyltransferase inhibitors (DNMTis) and histone deacetylase inhibitors (HDACis) are not always sufficiently selective and can disrupt essential gene expression programs in healthy tissues. This non-specific activity can result in adverse effects, such as hematological toxicity or gastrointestinal symptoms, restricting the safe use of these drugs.

Limited selectivity of some epigenetic inhibitors further raises concerns about both safety and efficacy. Broad-acting agents may inadvertently affect genes that are not directly involved in cancer, leading to unpredictable biological consequences and complicating clinical management. The development of next-generation inhibitors with greater specificity for cancer-associated epigenetic regulators is therefore a key research priority.

In summary, while targeting epigenetic alterations in cancer offers great promise, overcoming the challenges of tumor heterogeneity, epigenetic plasticity, off-target toxicity, and limited drug selectivity is essential for the successful clinical application of these therapies. Future research should focus on the development of highly selective agents, rational combination therapies, and biomarker-driven patient selection strategies to fully realize the potential of epigenetic interventions in oncology.

## Figures and Tables

**Table 1:** Clinically approved HDAC and DNMT inhibitors: cancer types, approval dates, and efficacy profiles.

Name of Drug	Type of Inhibitor	Cancer Type	Date of Approval	Efficacy Profile
**5-Azacitidine**	DNMT inhibitor	CMML, AML	2004 (US FDA approved)	Overall response rate (ORR)—13.9%
**Arsenic trioxide**	DNMT inhibitor	APL	2000 (US FDA approved)	Complete remission—100%
**Belinostat**	HDAC inhibitor	PTCL	2014 (US FDA approved)	Overall response rate (ORR)—25.8% Complete response rate (CR)—10.8% Partial response rate (PR)—15%
**Clofarabine**	DNMT inhibitor	AML	2004 (US FDA approved)	Overall response rate (ORR)—43%
**Decitabine**	DNMT inhibitor	MDS	2006 (US FDA approved)	Overall response rate (ORR)—61% Complete response rate (CR)—20% Overall survival (OS)—20 months
**Panobinostat**	HDAC inhibitor	MM	2015 (US FDA approved)	Objective response (OR)—58.5%
**Romidepsin**	HDAC inhibitor	CTCL	2009 (US FDA approved)	Overall response rate (ORR)—34% Complete response rate (CR)—6% Duration of response (DOR)—11/15 months
		PTCL	2011 (US FDA approved)	Overall response rate (ORR)—34% Complete response rate (CR)—15%
**Tucidinostat**	HDAC inhibitor	PTCL	2014 (NMPA approved)	Overall response rate (ORR)—29% Complete response rate (CR)—14%
**Vorinostat**	HDAC inhibitor	CTCL	2006 (US FDA approved)	Objective response (OR)—29.7% Average treatment duration (ATD)—5.3 months

**Table 2:** Classification of Epigenetic Drugs by Clinical Status (inhibitors undergoing preclinical/clinical trials selected).

Type of Inhibitors	Inhibitors Available for Patients	Inhibitors Undergoing Preclinical/Clinical Trials
**HDAC inhibitors**	Vorinostat Romidepsin Belinostat Panobinostat Tucidinostat (NMPA approved)	Abexinostat Fimepinostat Trichostatin A Entinostat KA2507 OBP-801 Givinostat
**BET inhibitors**		JQ1 JQ2 I-BET762 OTX015 ABBV-744
		SJ432 AZD5153 NHWD-870 ZL0513
**KDM4 inhibitors**		N-oxalylglycine (NOG) Pyridinedicarboxylic Acid (PCA) 8-hydroxyquinoline (8-HQ) TACH101 2-(1H-tetrazol-5-yl) acetohydrazid JIB-04 NSC636819
**DNMT inhibitors**	5-Azacitidine Decitabine Clofarabine Arsenic trioxide	Guadecitabine RX-3117 5-Fluoro-2′-deoxycytidine Fazarabine Cladribine and fludarabine Procaine Epigallocatechin gallate Hydralazine Genistein and equol Curcuminum Disulfiram Resveratrol Caffeic acid
**NNMT inhibitors**		GYZ-319 Compound 17u Macrocyclic peptides (peptide 4 and 13) 6-MANA

## Data Availability

Not applicable.

## Abbreviations

2-OG	2-oxoglutarate
8-HQ	8-hydroxyquinoline
AML	Acute myeloid leukemia
APC	Antigen-presenting cells
APL	Acute promyelocytic leukemia
ARID1A	AT-Rich Interaction Domain Containing protein 1A
ARID1B	AT-Rich Interaction Domain Containing protein 1B
ASCT	Autologous stem-cell transplantation
BAF	Barrier-to-autointegration factor
BC	Breast cancer
BER	Base excision repair
BET	Bromodomain and extra-terminal domain
BETi	BET protein inhibitors
biBET	Bivalent BET protein inhibitor
BRCA2	Breast Cancer 2
CBP	CREB-binding protein
CDKN2A	Cyclin-Dependent Kinase Inhibitor 2A
CGIs	CpG islands
CHD	Chromodomain helicase DNA-binding
circRNA	Circular RNA
CpG	C–phosphate–G
CR	Complete response
CRC cells	Colorectal Cancer cells
CREB	cAMP response element-binding protein
CSF-1	Colony-stimulating factor
CTCL	Cutaneous T-cell lymphoma
CTL	Cytotoxic T lymphocytes
DNA-PKcs	DNA-dependent protein kinase catalytic subunit
DNMT1	DNA (cytosine-5)-methyltransferase 1
DNMT3A	DNA (cytosine-5)-methyltransferase 3 alpha
DNMT3B	DNA (cytosine-5)-methyltransferase 3 beta
DNMTi	DNA methyltransferase inhibitors
DNMTs	DNA methyltransferases
DOR	Duration of response
DSB	Double-strand breaks
EGFR-TKI	Epidermal growth factor receptor tyrosine kinase inhibitors
EZH1	Enhancer of Zeste Homolog 1
EZH2	Enhancer of Zeste Homolog 2
FDA	United States Food and Drug Administration
FGFR1	Fibroblast Growth Factor Receptor Protein
GBM	Glioblastoma
GCN5	General control non-repressed protein 5
GI50	Half maximal growth inhibition
GNAT	GCN5-related N-acetyltransferases
H3K27me3	Histone H3 Lysine 27 Trimethylation
H3K36me2	Histone H3 Lysine 36 Dimethylation
H3K36me3	Histone H3 Lysine 36 Trimethylation
H3K4me3	Histone H3 Lysine 4 Trimethylation
H3K9me3	Histone H3 Lysine 9 Trimethylation
HATs	Histone acetyltransferases
HCC	Hepatocellular carcinoma
HDACi	Histone deacetylase inhibitors
HDAC	Histone deacetylases
HDM	Histone demethylases
HL	Hodgkin’s lymphoma
HMT	Histone methyltransferases
HNSCC	Head and neck squamous cell carcinoma
HOXB-AS3	HOXB Cluster Antisense RNA 3
HR	Homologous recombination
IC50	Half maximal inhibitory concentration
ICD	Immunogenic cell death
INO80	Inositol requiring 80
ISWI	Imitation Switch
JmjC	Jumonji C
KDM	Histone lysine demethylases
lncRNAs	Long non-coding RNAs
LSD1	Lysine-specific demethylase 1
MAPK1	Mitogen-Activated Protein Kinase 1
MBD1	Methyl-CpG-binding domain protein 1
MBD2	Methyl-CpG-binding domain protein 2
MBD3	Methyl-CpG-binding domain protein 3
MBDs	Methyl-CpG Binding Domain
MDS	Myelodysplastic syndrome
MeCP2	Methyl-CpG-binding protein 2
MHC-I	Major histocompatibility complex I
miRNAs	MicroRNAs
MM	Multiple myeloma
NAD+	Nicotinamide adenine dinucleotide
ncRNA	Non-coding RNA
NHEJ	Non-homologous end joining
NK	Natural killer
NMC	NUT midline carcinoma
NOG	N-oxalylglycine
NuRD	Nucleosome remodeling and deacetylase
NSCLC	Non-small cell lung cancer
NSD1	Nuclear receptor SET domain-containing protein 1
NSD2	Nuclear receptor SET domain-containing protein 2
NSD3	Nuclear receptor SET domain-containing protein 3
ORR	Overall response rate
PARP	Poly (ADP-ribose) polymerase
PBRM1	Protein Polybromo-1 gene
PCA	Pyridinedicarboxylic acid
PFS	Progression-free survival
PHD	Double prolyl hydroxylase
PR	Partial response
PRC2	Polycomb Repressive Complex 2
pri-miRNA	Primary microRNA
PTCL	Peripheral T-cell lymphoma
RECIST	Response Evaluation Criteria in Solid Tumors
RNAPII	RNA polymerase II
SAM	S-adenosyl methionine
SDSA	Synthesis-dependent strand annealing
SET	Su(var)3-9, Enhancer of zeste [E(z)], Trithorax
siRNA	Small interfering RNA
SIRT	Sirtuin
SMARCA2	SWI/SNF Related, Matrix Associated, Actin Dependent Regulator of Chromatin, Subfamily A, Member 2
SMARCA4	SWI/SNF Related, Matrix Associated, Actin Dependent Regulator of Chromatin, Subfamily A, Member 4
sORFs	Small open reading frames
ssDNA gaps	Single-stranded DNA gaps
SWI/SNF	Switch/Sucrose Non-Fermentable
TAA	Tumor-associated antigen
TAM	Tumor-associated macrophages
TET2	Ten-Eleven Translocation 2
TSG	Tumor suppressor gene
TTR	Time to response
VGPR	Very good partial response
XAS	X-ray Absorption Spectroscopy
